# Supplementary figures and images for: Neuronal cell cycle reentry events in the aging brain are more prevalent in neurodegeneration and lead to cellular senescence
Source: PLoS Biol. 2024 Apr 23;22(4):e3002559. doi: 10.1371/journal.pbio.3002559 (PMC11037540; doi:10.1371/journal.pbio.3002559)

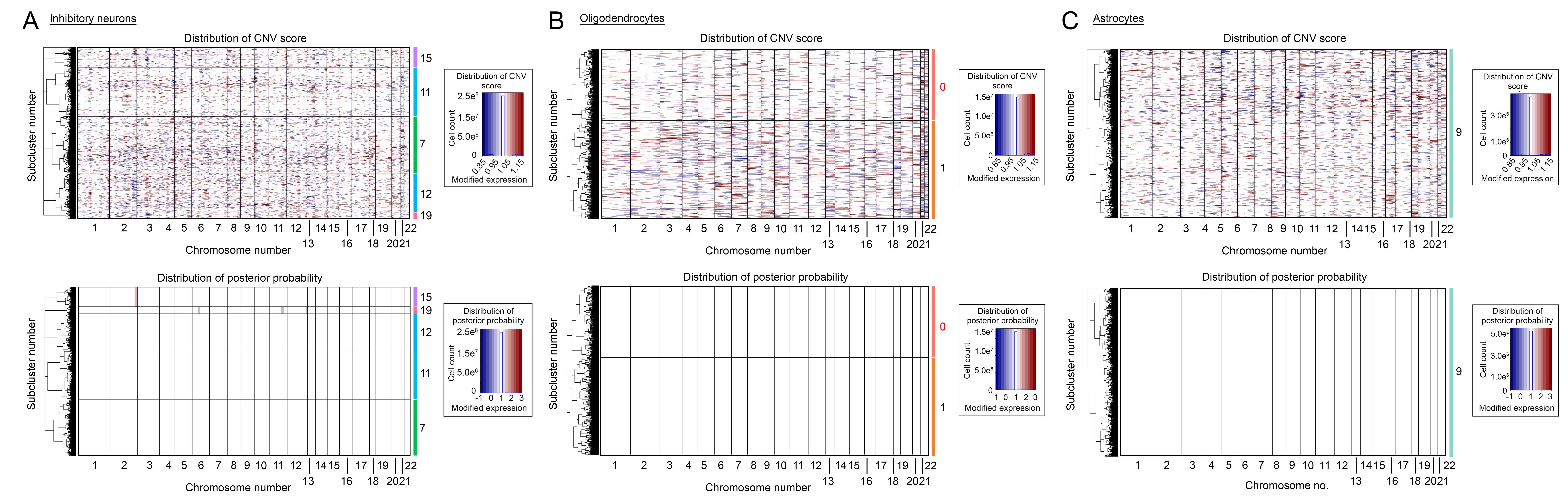

Supplement: S1 Fig — Estimation of copy number variants by the InferCNV algorithm in (A) inhibitory neurons, (B) oligodendrocytes, and (C) astrocytes. The heatmap located at the top of each panel indicates the copy number alteration regions identified by the hidden Markov model, i.e., regions of gain (red) and loss (blue) in expression along each chromosome at various regions from the p-arm (left side of each box) to the q-arm (right side of each box), in all subclusters. The heatmap located at the bottom of each panel is an outcome of the Bayesian latent mixture model implemented to identify the posterior probabilities of alteration status in each cell and whole CNA region. Red: gain of copy number. Blue: loss of copy number. (JPG) [file pbio.3002559.s001.jpg]

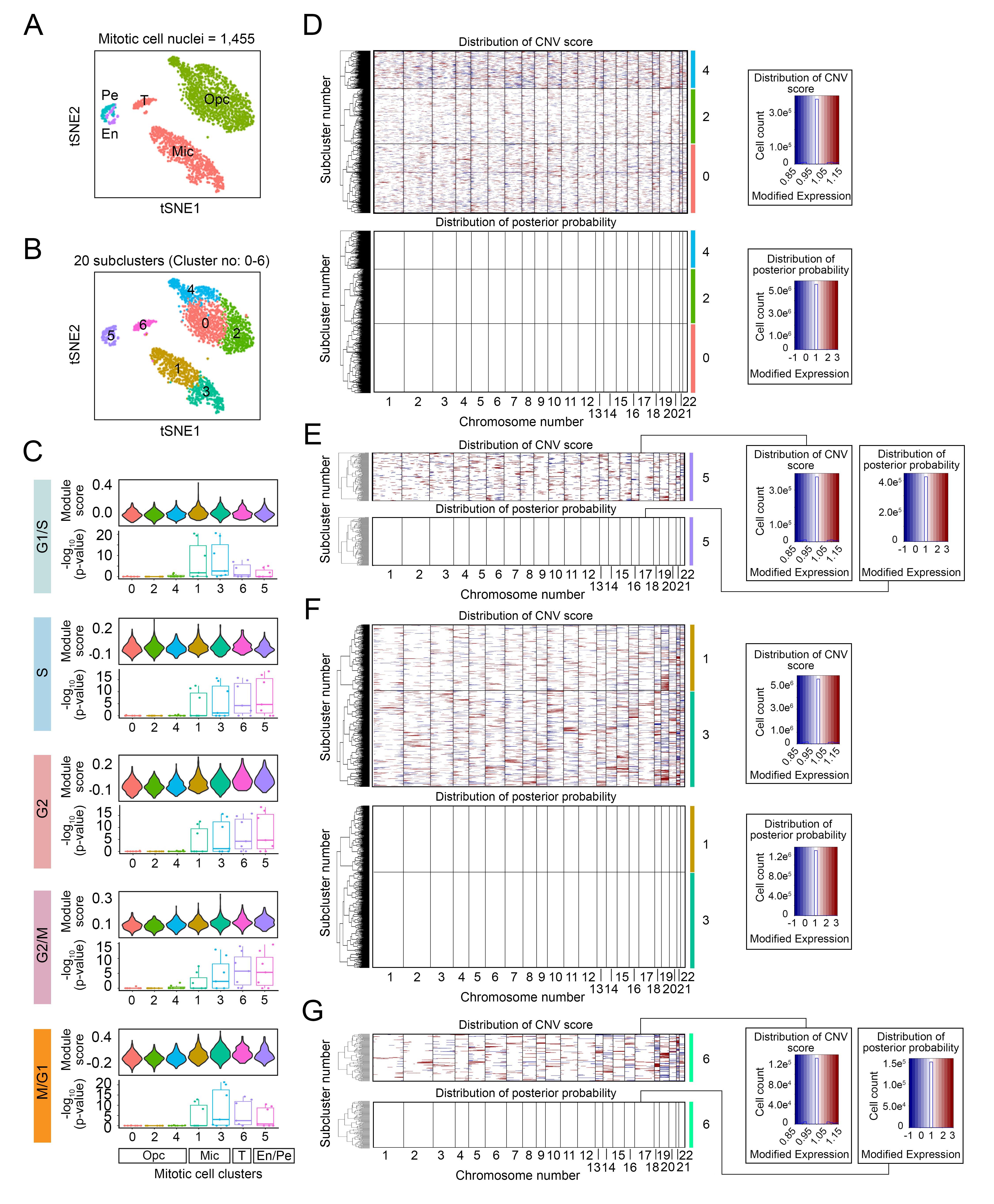

Supplement: S2 Fig — (A, B) t-SNE plot of 1,455 mitotic cell nuclei. (A) Cell types are colored differently: microglia (Mic), endothelial cells (En), oligodendrocyte progenitor cells (Opc), pericytes (Per), and T cells (T). (B) Cells are colored based on subclustering numbers. (C) Violin plots of the cell cycle phase scores of all subclusters of mitotic nuclei. Bolded violins in different phases indicate the subcluster with the most significant above-average gene expression levels among all the clusters. Corresponding significance values against other clusters are shown below. (D–G) Estimation of copy number variants by the InferCNV algorithm in (D) Opc, (E) En/Per, (F) Mic, and (G) T cells. The heatmap located at the top of each panel indicates the copy number alteration regions identified by the hidden Markov model, e.g., regions of gain (red) and loss (blue) in expression along each chromosome at various regions from the p-arm (left side of each box) to the q-arm (right side of each box), in all subclusters. The heatmap located at the bottom of each panel is an outcome of the Bayesian latent mixture model implemented to identify the posterior probabilities of alteration status in each cell and whole CNA region. Red: gain of copy number. Blue: loss of copy number. The metadata underlying this figure can be found at https://zenodo.org/doi/10.5281/zenodo.10604562. (JPG) [file pbio.3002559.s002.jpg]

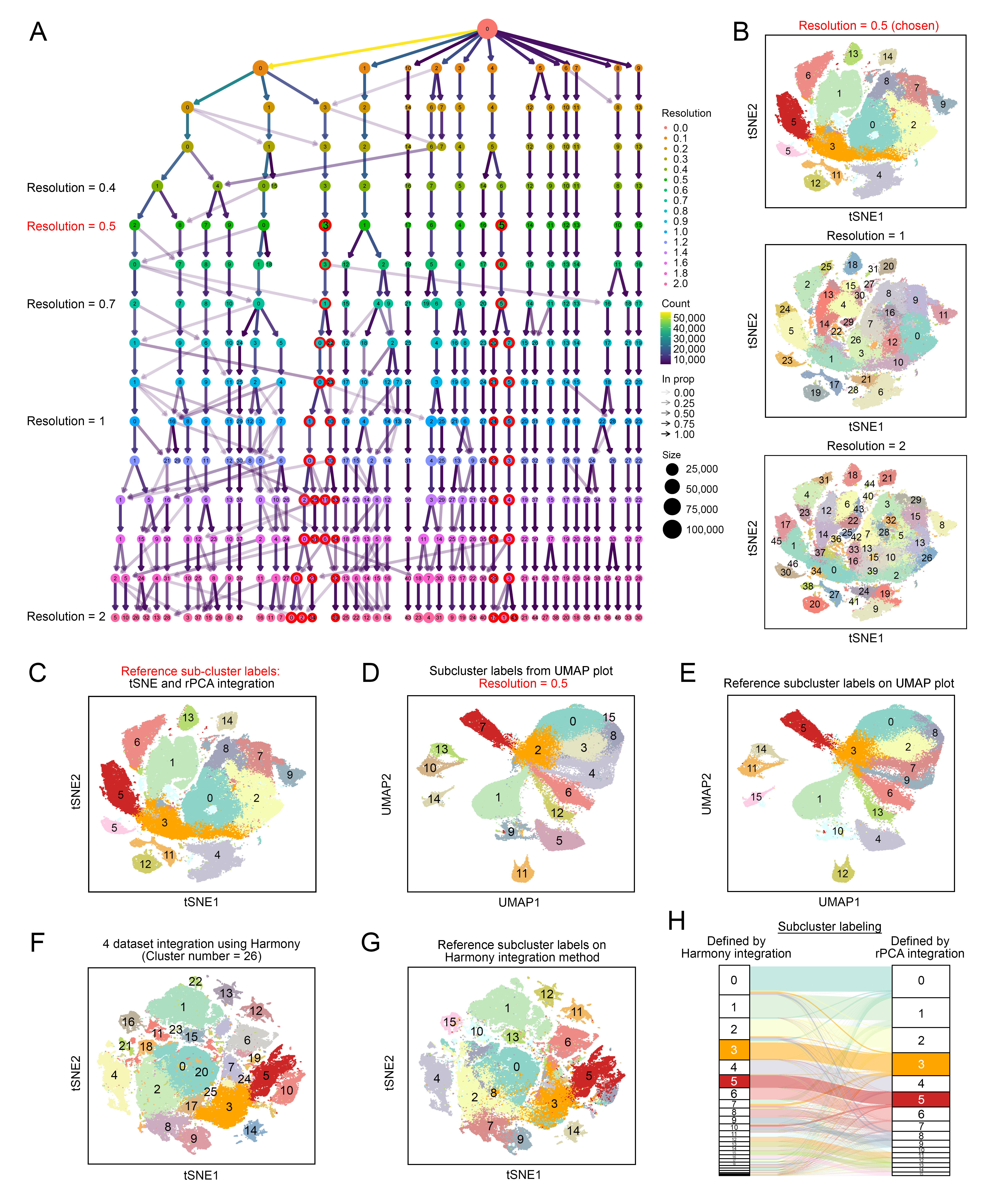

Supplement: S3 Fig — (A) Clustering tree diagram illustrating how excitatory neuronal subclusters were defined at different resolution settings. Each row represents the number of subclusters identified at one particular resolution, and each column represents the number of subclusters and consistencies across different resolutions. As expected, higher numbers of subclusters were defined under higher resolution settings. The target subclusters (e.g., #3 and #5) had already emerged since the resolution was set at 0.4, and their distinct identities held when the settings were increased to 0.7. As the resolution increased to greater than 0.7, these 2 major subclusters started to split into smaller subclusters; nevertheless, they never cross-mixed with others derived from non-cell cycle re-engaging neurons and vice versa. (B) t-SNE plots of excitatory neuronal nuclei subclustering in the 4-dataset integrated analysis settings at different resolutions corresponding to those illustrated in (A). (C) t-SNE and (D) UMAP plots of excitatory neuronal subclusters generated by the rPCA integration method at a resolution of 0.5. (E) UMAP plot of the excitatory neuronal subclusters generated by the t-SNE dimension reduction method. (F) t-SNE plot of the excitatory neuronal subclustering generated by the Harmony integration method at a resolution of 0.5. (G) Excitatory neuronal nuclei were colored and labeled according to the findings from the rPCA integration method, as illustrated in (C). (H) Alluvial diagram showing the intersecting subclusters defined by the Harmony (left) versus the rPCA (right) integration methods. The resulting high consistency in subcluster identity indicated that excitatory neuronal cluster identification was not affected by the integration methods or other analytical settings. The metadata underlying this figure can be found at https://zenodo.org/doi/10.5281/zenodo.10604562. (TIF) [file pbio.3002559.s003.tif]

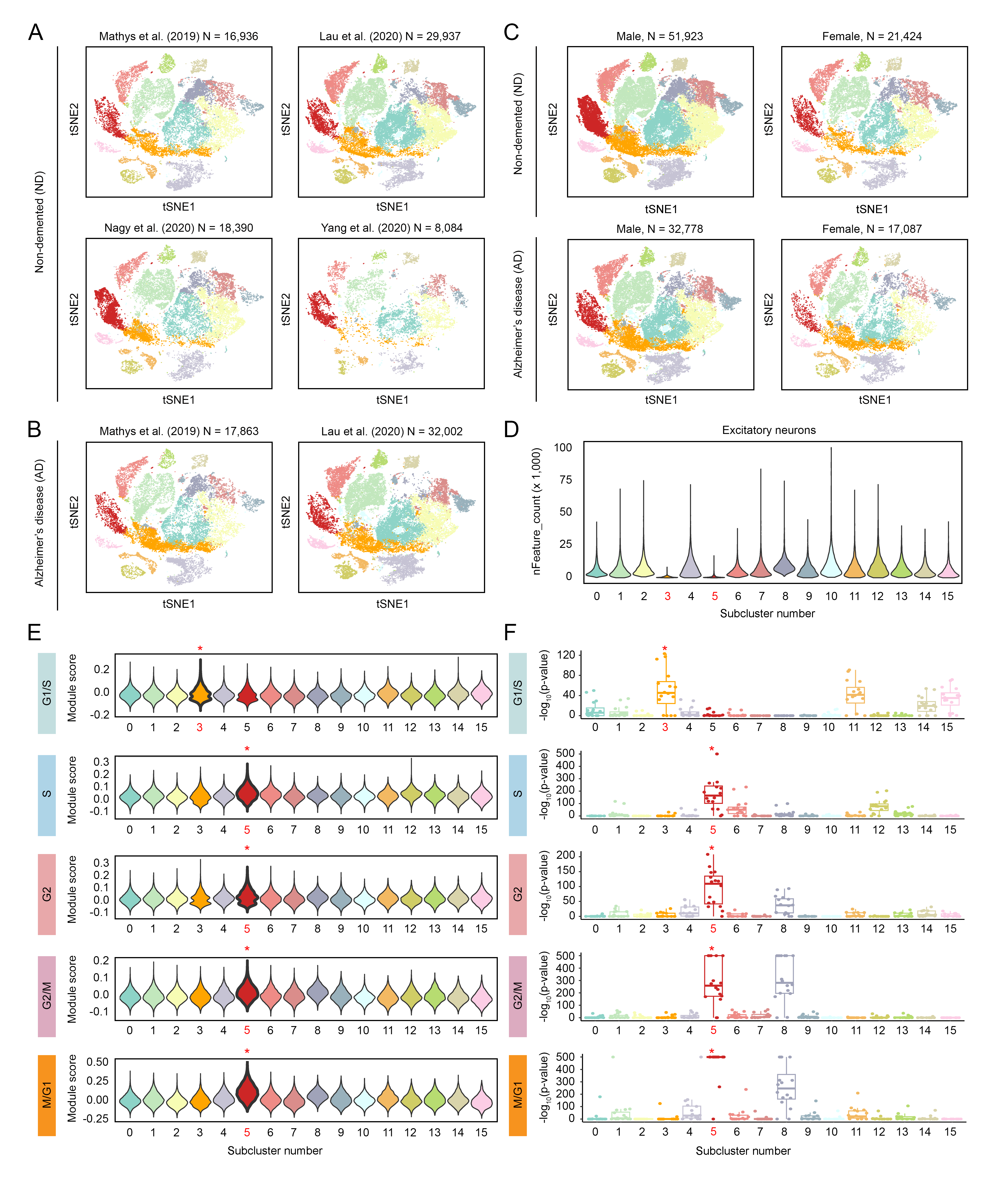

Supplement: S4 Fig — (A) t-SNE plots of excitatory neuronal nuclei extracted from nondemented (ND) samples from different studies. (B) t-SNE plots of excitatory neuronal nuclei extracted from disease-affected (AD) samples from Mathys and colleagues and Lau and colleagues. (C) t-SNE plots of the excitatory neuronal nuclei distribution based on sex and disease status. (D) Violin plot illustrating the average feature counts of global transcriptomic profiles among excitatory neurons in different subclusters. (E) Violin plots presenting the cell cycle phase scores of all subclusters of excitatory neurons. Bolded violins highlighted in different phases indicate the subclusters that exhibit the most significant above-average cell cycle gene reexpression among all the subclusters. The corresponding significance values obtained for each subcluster compared to the rest of the others are shown in (F). The metadata underlying this figure can be found at https://zenodo.org/doi/10.5281/zenodo.10604562. (TIF) [file pbio.3002559.s004.tif]

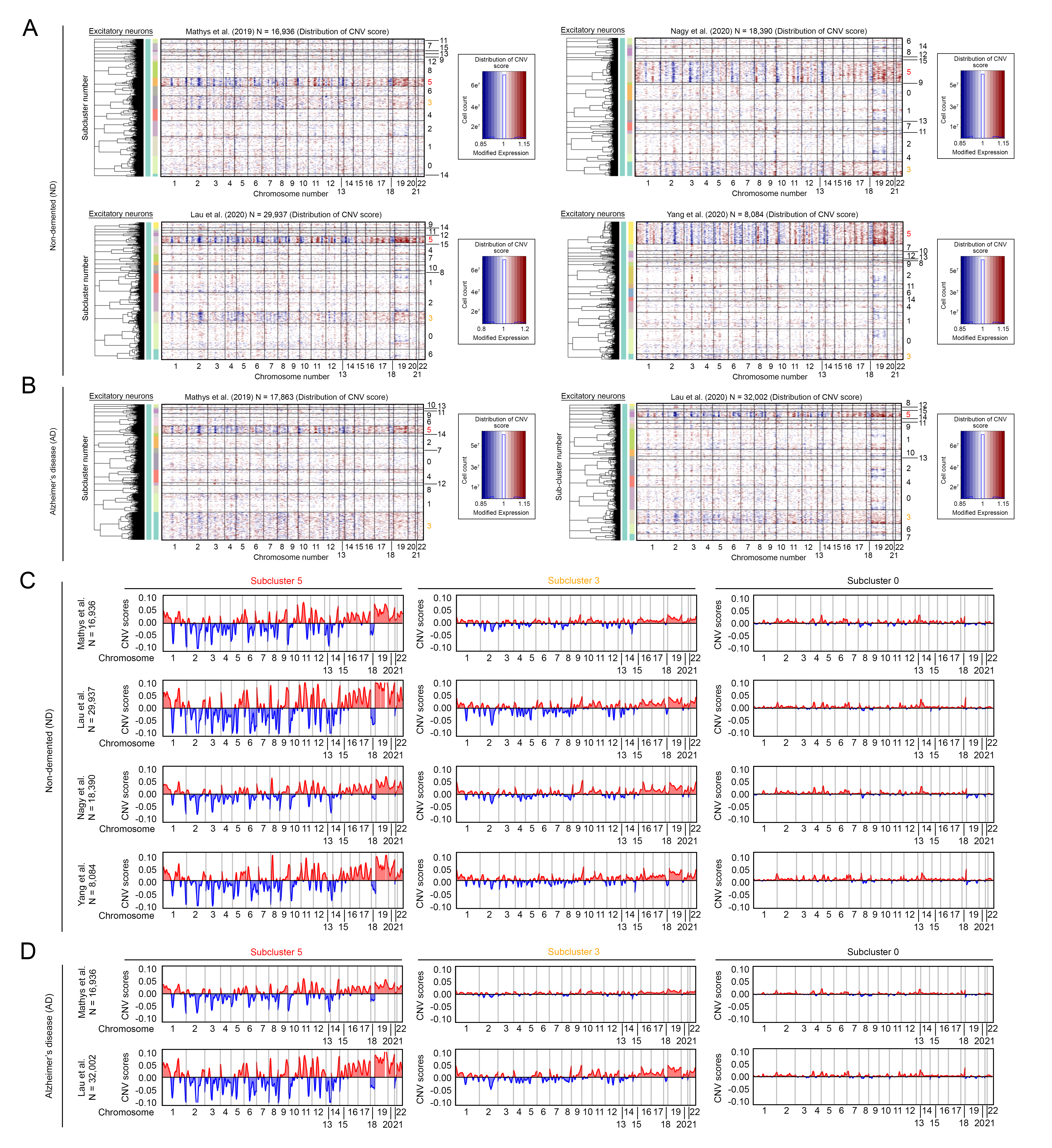

Supplement: S5 Fig — (A, B) Estimation of copy number variants in excitatory neuronal clusters extracted from either (A) nondemented (ND) or (B) disease-affected (AD) brain samples by the InferCNV algorithm. Red: gain of copy number. Blue: loss of copy number. (C, D) Line plots illustrating in detail how gene expression levels at different chromosome locations were altered in subclusters 3 and 5 compared to those in the negative control subcluster 0 extracted from either (C) nondemented (ND) or (D) disease-affected (AD) brain samples. The metadata underlying this figure can be found at https://zenodo.org/doi/10.5281/zenodo.10604562. (TIF) [file pbio.3002559.s005.tif]

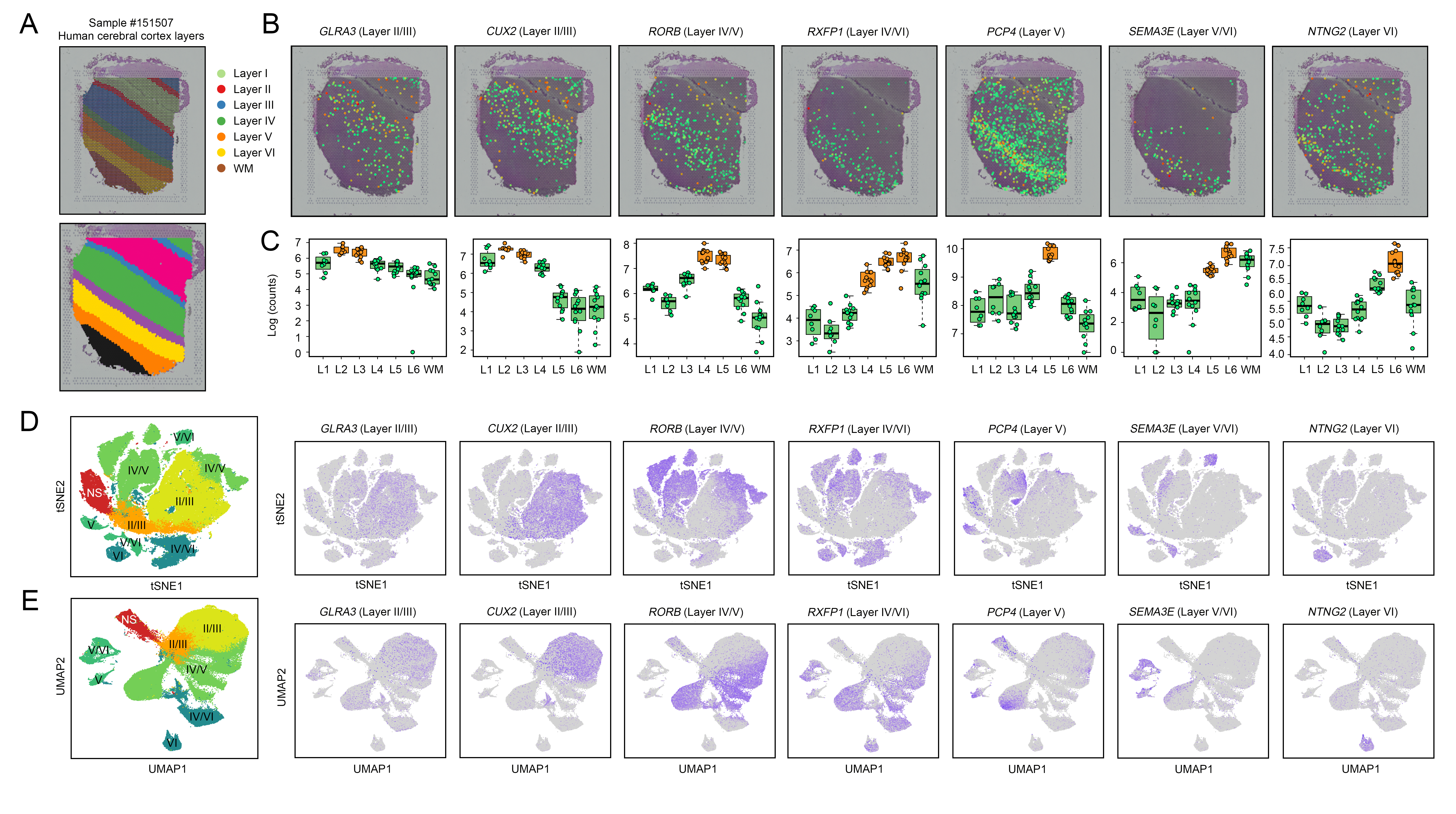

Supplement: S6 Fig — (A) Visualization of cortical layers in sample #151707 from the jhpce#HumanPilot10x dataset using spatialLIBD. (B) Visualization of the distribution and counts of cortical layer-specific markers per spot. (C) Boxplots showing the expression levels of various layer-specific markers across different spatial locations defined in (A) to validate their layer specificities. (D) t-SNE and (E) UMAP plots illustrating the differential enrichment of cortical layer-specific markers in different neuronal subclusters in the integrated cohort analyses. (TIF) [file pbio.3002559.s006.tif]

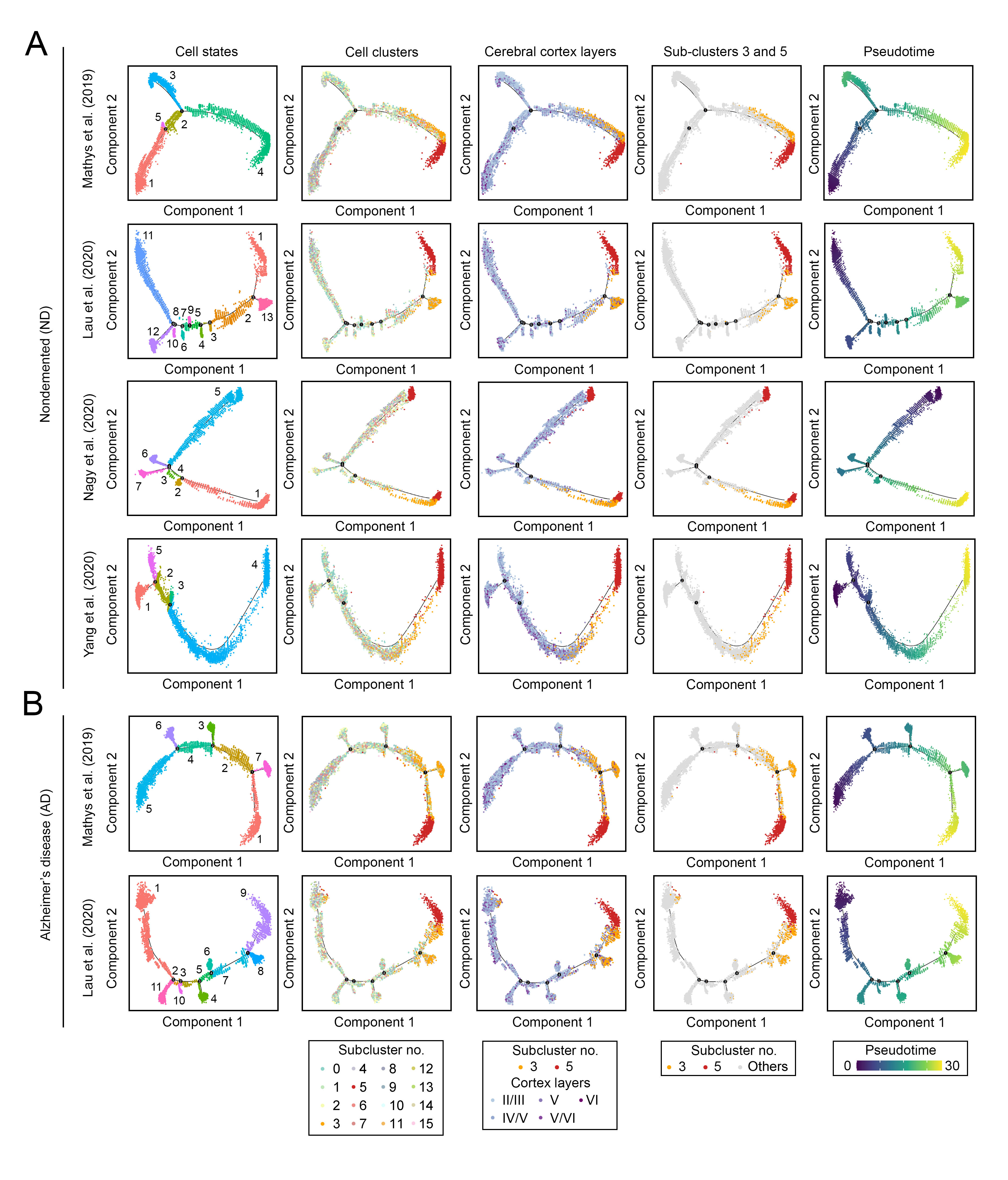

Supplement: S7 Fig — Cells on the trees are colored based on cell states, subcluster identities, cortical layer distributions, senescent neuronal cluster assignments, and pseudotime scales. Subclusters 3 and 5 were found along the same branch and were deemed to be the most similar to one another based on their pseudotime values. Subcluster 3, located at the terminal location of a branch, indicated terminal cell fate. The data represent findings from (A) nondemented (ND) or (B) disease-affected (AD) samples. (TIF) [file pbio.3002559.s007.tif]

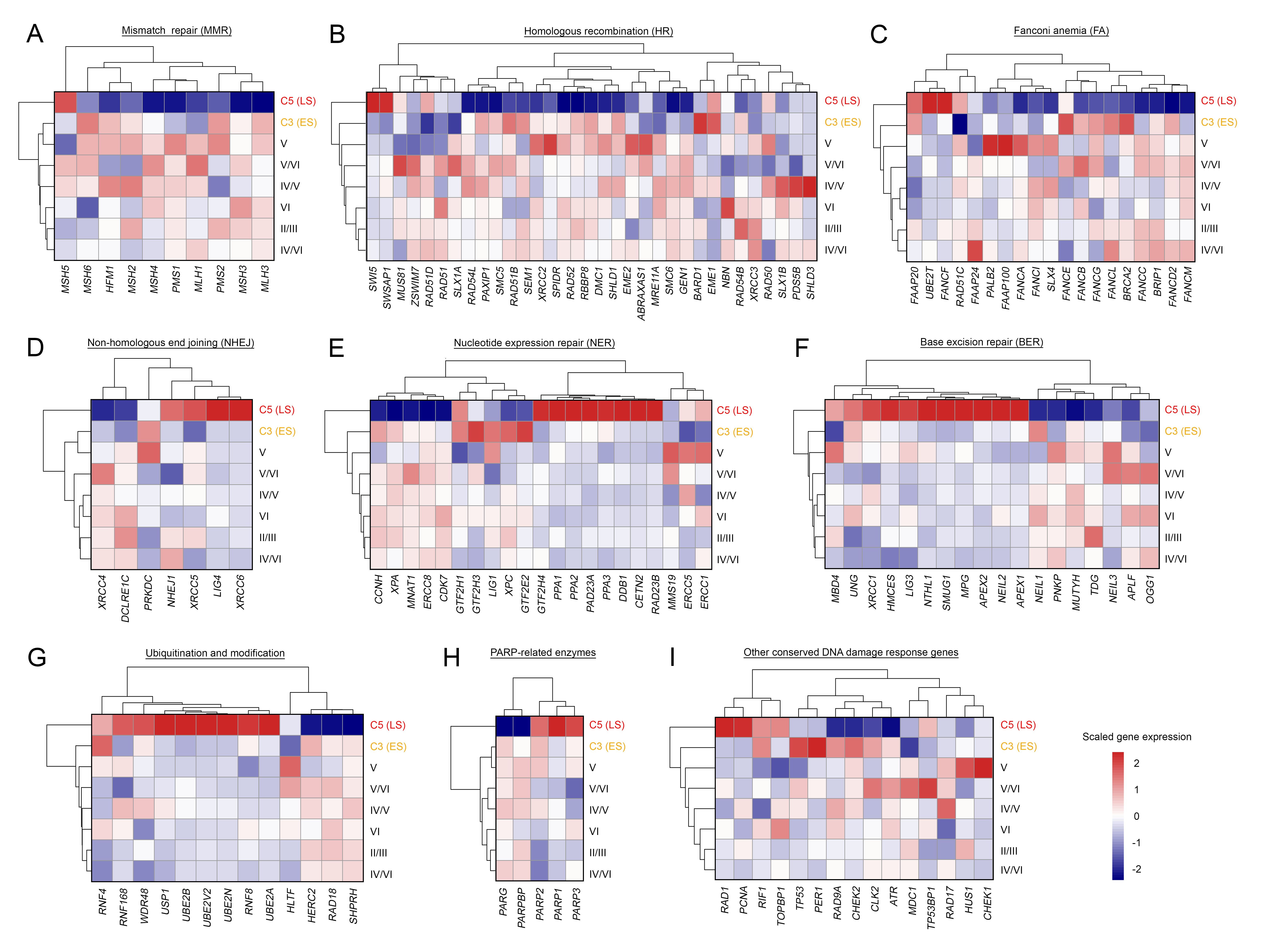

Supplement: S8 Fig — (A–I) Normalized expression levels of core DNA damage response genes implicated in (A) mismatch repair, (B) homologous recombination, (C) Fanconi anemia, (D) nonhomologous end joining, (E) nucleotide excision repair, (F) base excision repair, (G) ubiquitination and modification, (H) poly(ADP-ribose) polymerase (PARP) enzymes that bind to DNA, and (I) other conserved DNA damage response genes. The metadata underlying this figure can be found at https://zenodo.org/doi/10.5281/zenodo.10604562. (TIF) [file pbio.3002559.s008.tif]

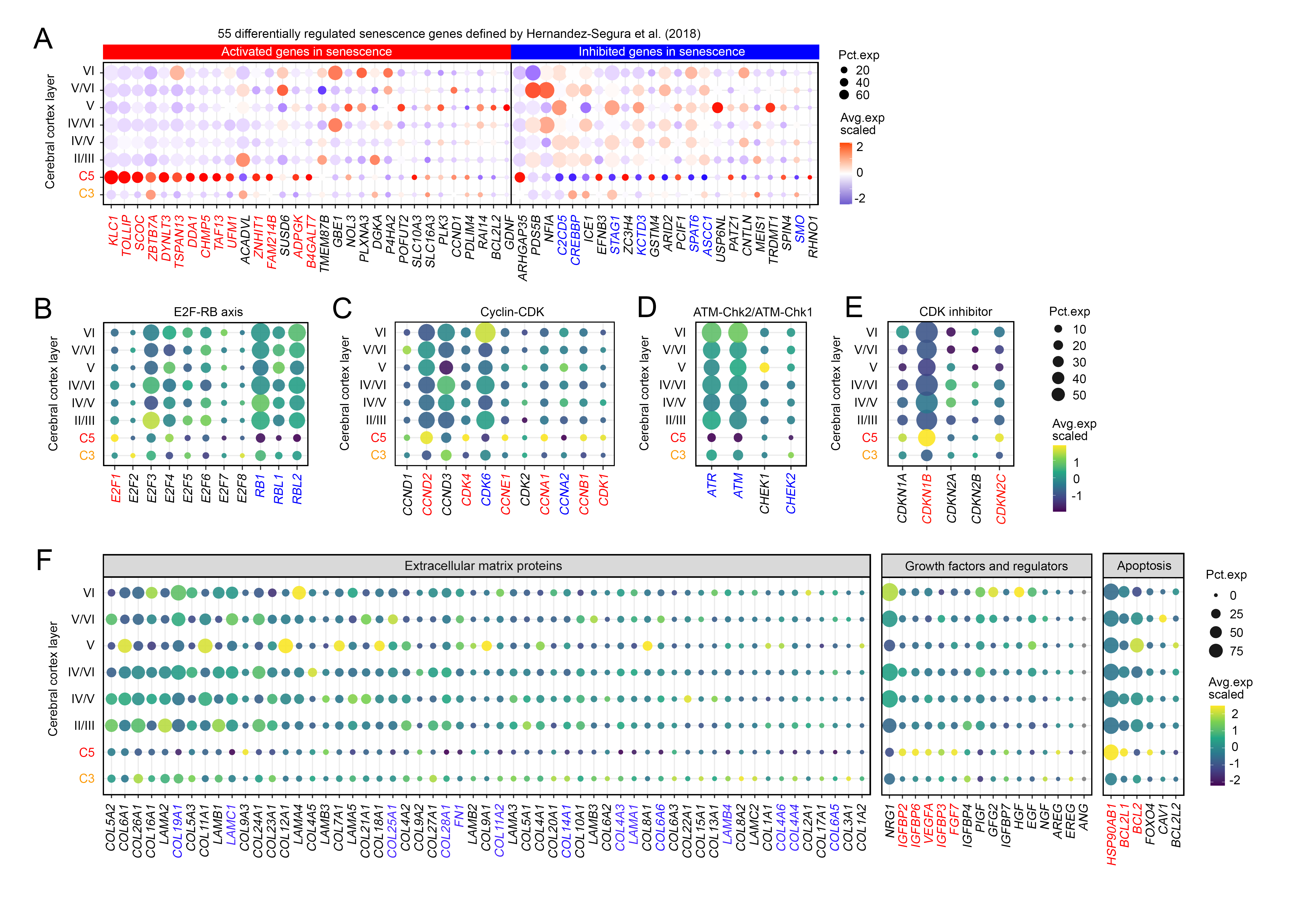

Supplement: S9 Fig — (A) Expression levels of 55 differentially regulated senescence genes defined by the Hernandez-Segura and colleagues study [47]. (B–F) Expression levels of selected and classic genes involved in the (B) E2F-RB axis, (C) Cyclin-CDK axis, (D) ATM-Chk2/ATM-Chk1 axis, (E) CDK inhibitor, and (F) senescence-associated secretory phenotype profile obtained from the study by Coppe and colleagues [102]. The metadata underlying this figure can be found at https://zenodo.org/doi/10.5281/zenodo.10604562. (TIF) [file pbio.3002559.s009.tif]

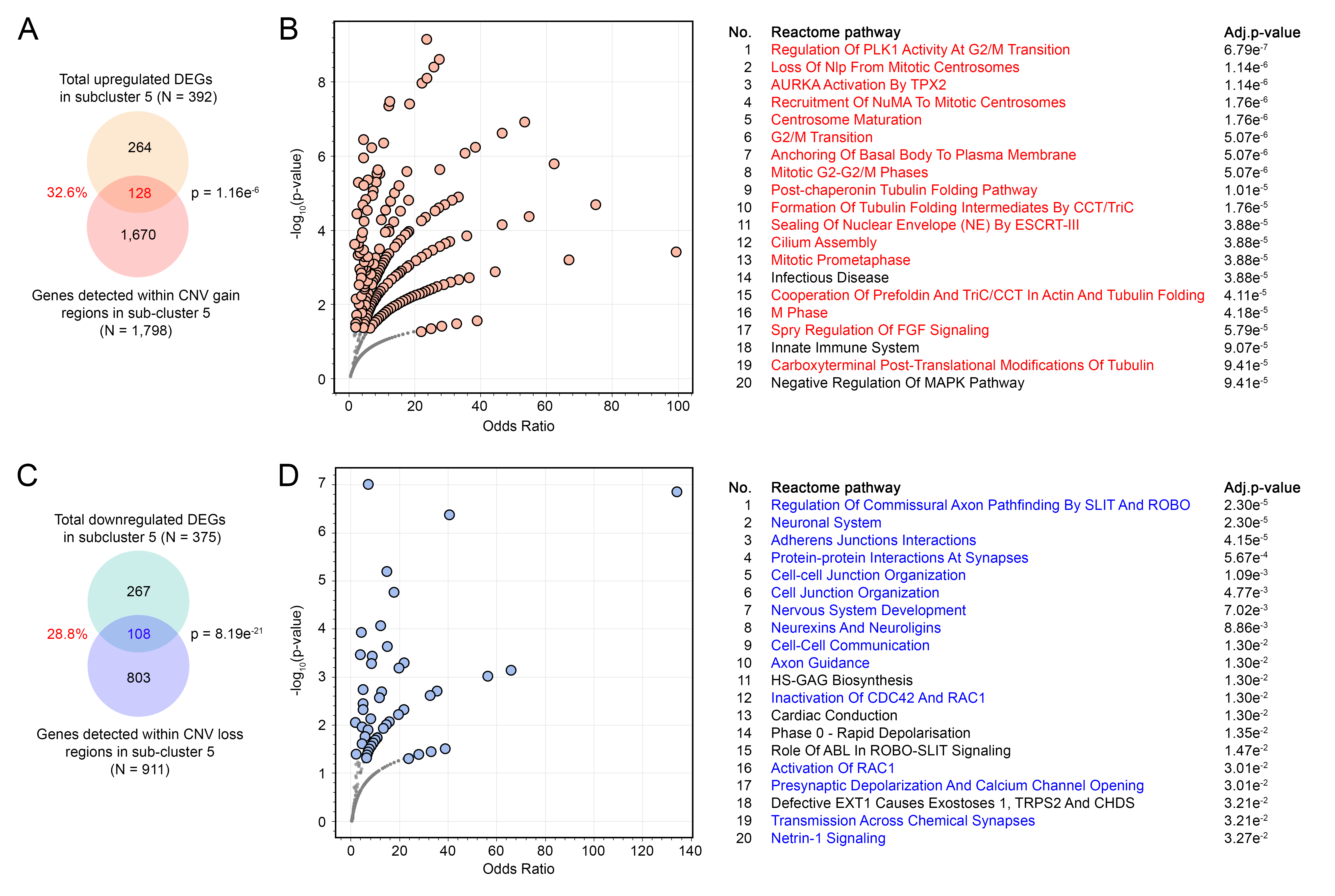

Supplement: S10 Fig — (A, C) Venn diagram illustrating the degree of similarity of the DEGs identified in subcluster 5 compared to the remaining non-cell cycle gene reexpressing excitatory neurons in the list of genes located in the predicted (A) CNV gain and (C) loss regions. (B, D) Functional overrepresentation analysis of common genes identified in (A) and (C), respectively, with reference to pathways in the Reactome database. The metadata underlying this figure can be found at https://zenodo.org/doi/10.5281/zenodo.10604562. (TIF) [file pbio.3002559.s010.tif]

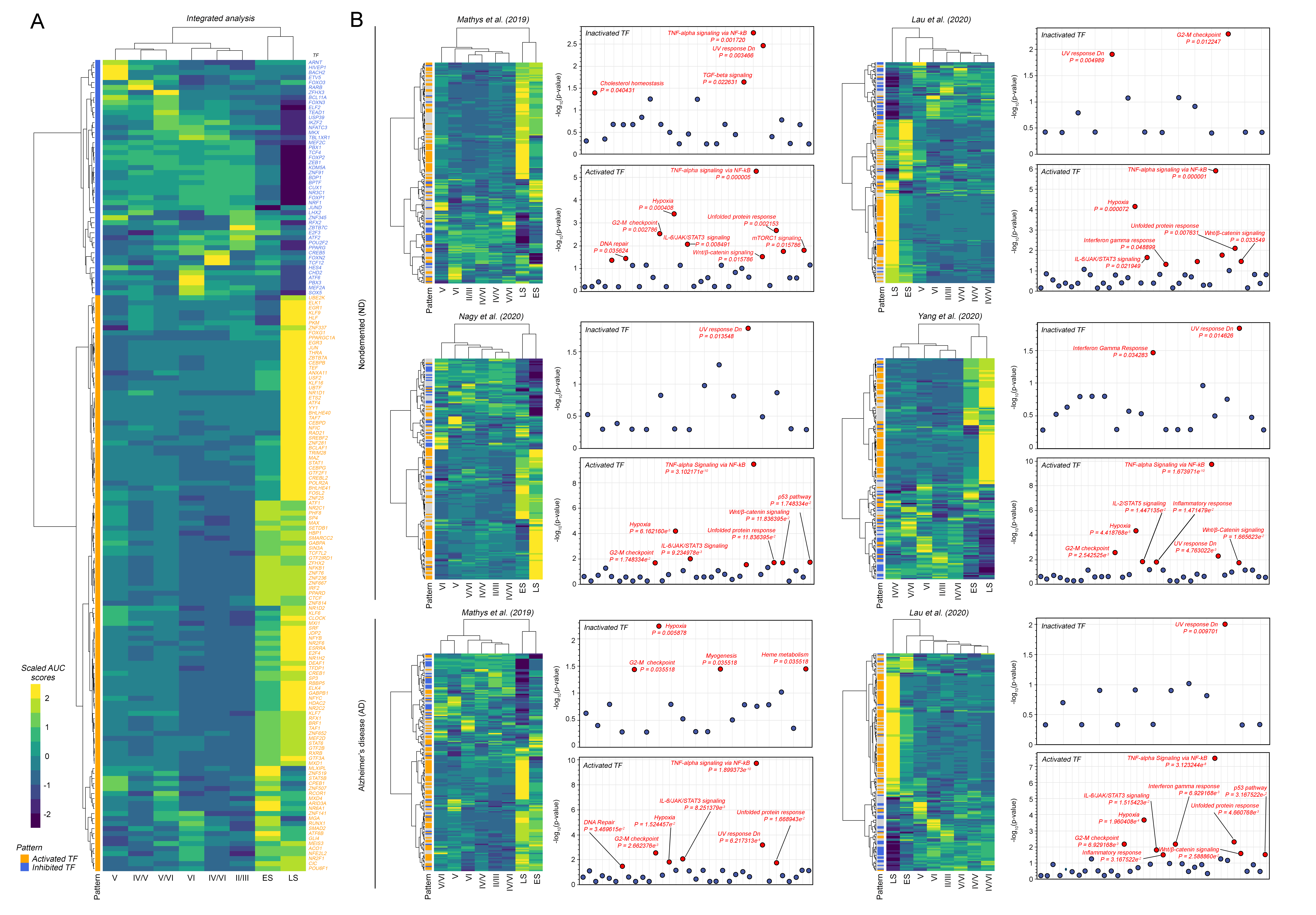

Supplement: S11 Fig — (A) In an integrated cohort setting, an SCENIC binary regulon activity matrix showing that all 162 corrected regulons were activated in more than one subcluster. Each column represents neurons in a single cortical layer (or ES or LS neuronal clusters), and each row represents one regulon. The term “regulon” refers to the regulatory network of transcription factors and their target genes. Key regulons (rows) are magnified and colored according to their activities: active (orange) or inactive (blue) in the ES and LS neuronal clusters. (B) The same set of analyses illustrated in (A) was conducted separately for each individual dataset. Manhattan plots illustrating the enriched signaling networks of coinhibited (top panel) or coactivated (bottom panel) TFs. Significantly (p < 0.05) enriched networks are labeled. The metadata underlying this figure can be found at https://zenodo.org/doi/10.5281/zenodo.10604562. (TIF) [file pbio.3002559.s011.tif]

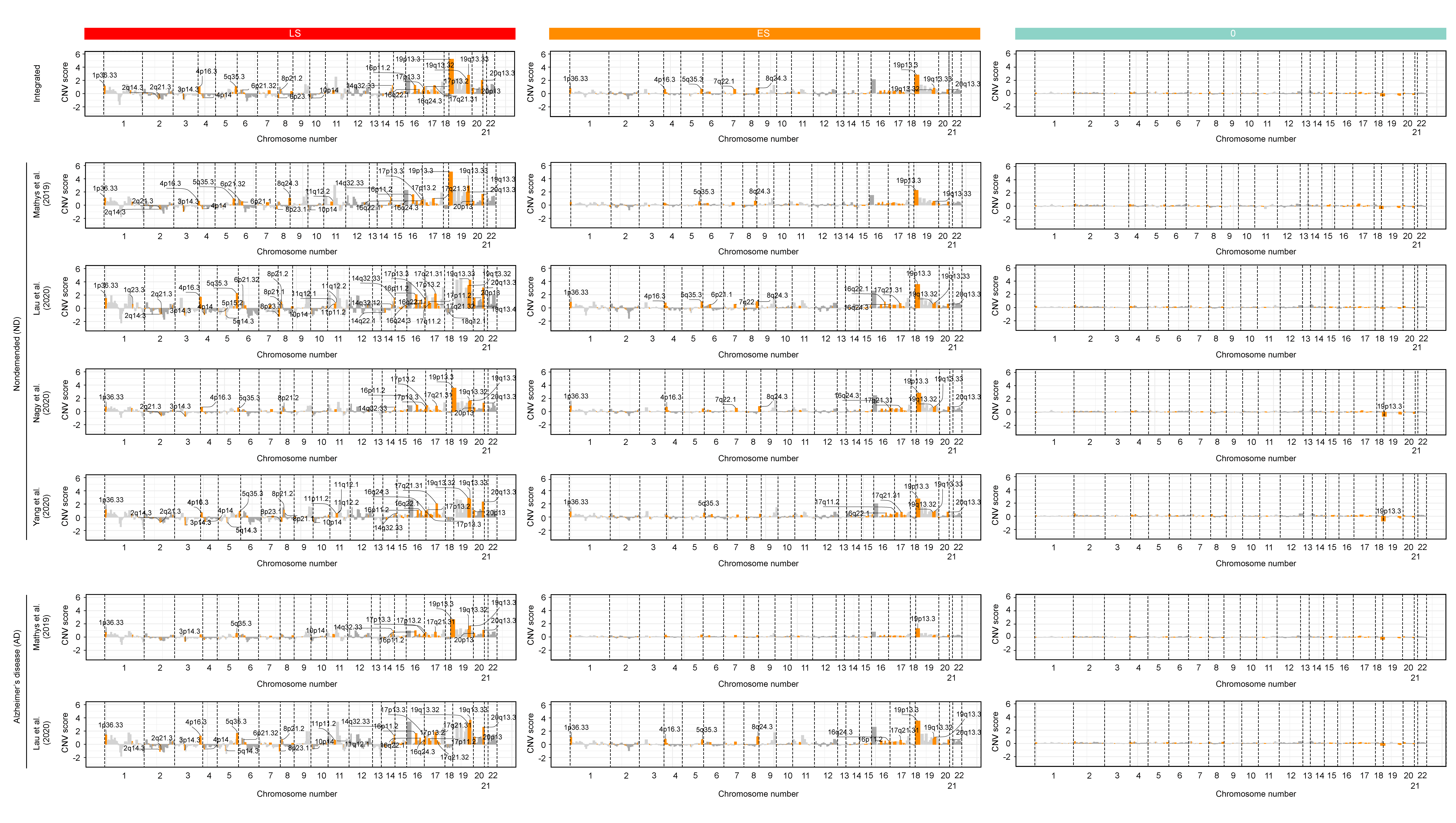

Supplement: S12 Fig — The yellow highlights indicate chromosomal locations of classic AD risk gene loci. The metadata underlying this figure can be found at https://zenodo.org/doi/10.5281/zenodo.10604562. (TIF) [file pbio.3002559.s012.tif]

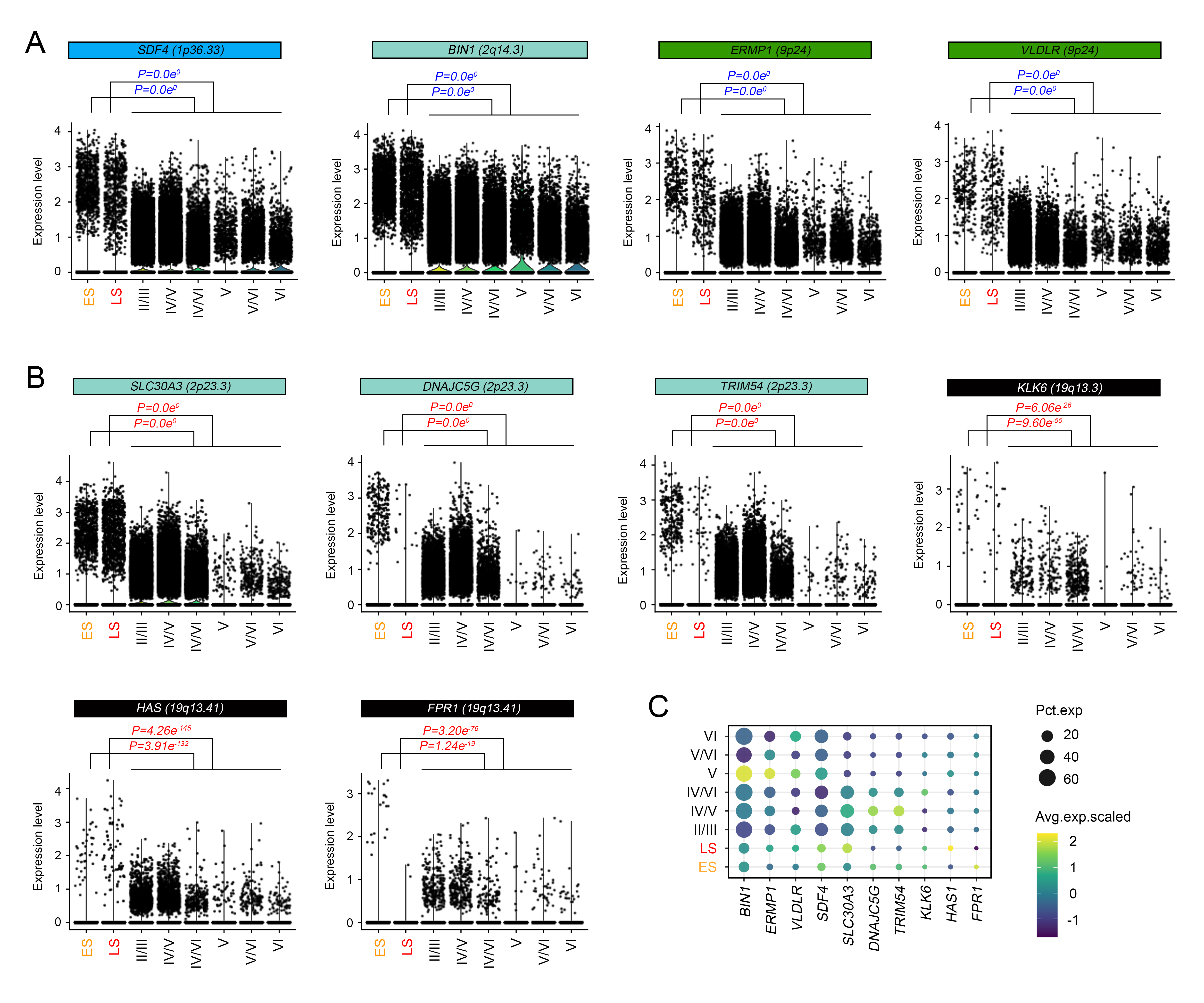

Supplement: S13 Fig — Comparisons were made among ES, LS, and the remaining non-cell cycle re-engaging neurons stratified based on their cortical layer identity. Reference list of CNV genes that were found to be (A) lost or (B) gained in AD. (C) Dot plot presenting the scale average expression levels of these canonical AD-related CNV genes among all the clusters compared. The metadata underlying this figure can be found at https://zenodo.org/doi/10.5281/zenodo.10604562. (TIF) [file pbio.3002559.s013.tif]

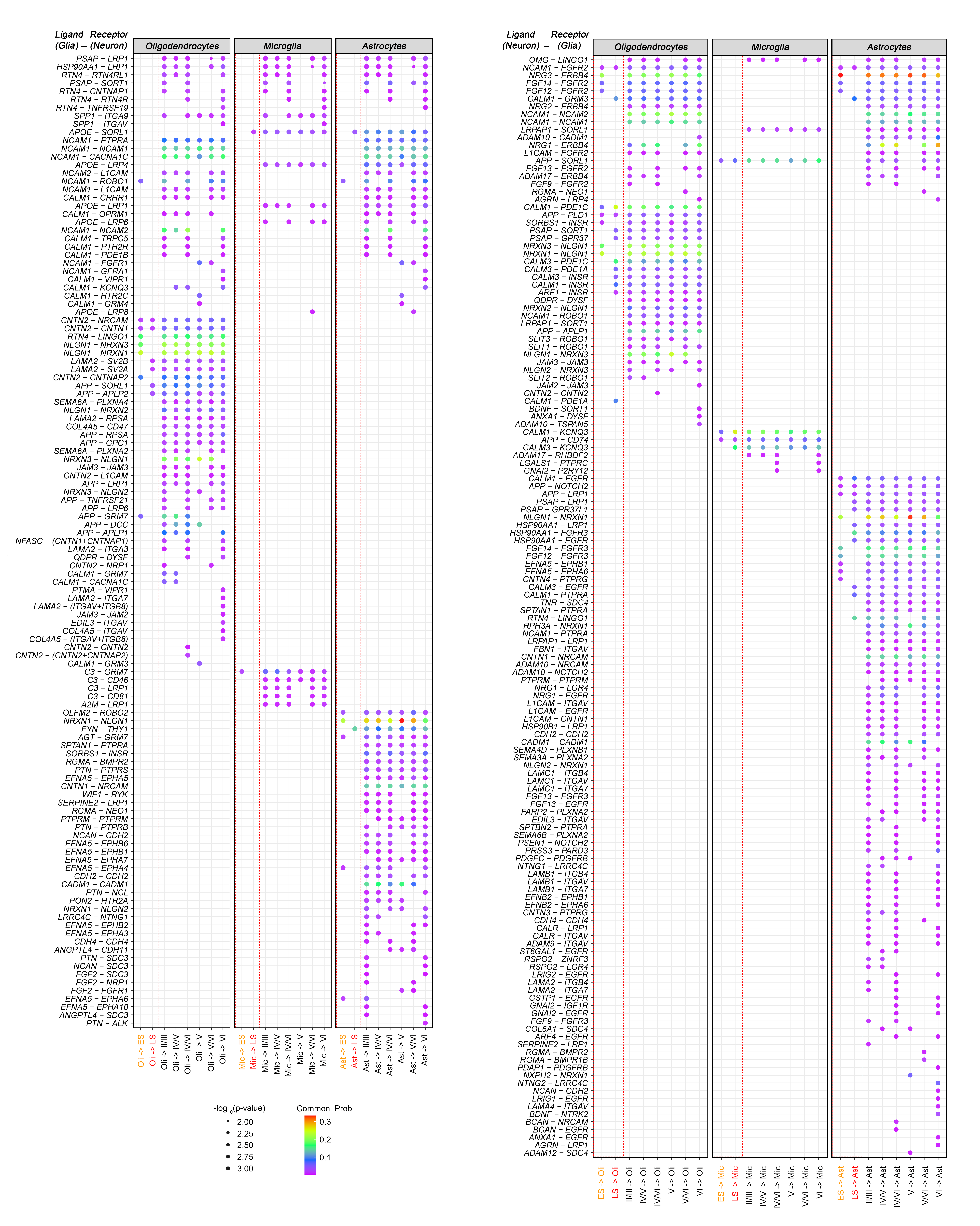

Supplement: S14 Fig — Dot plots illustrating the detailed results of the connectome analysis, which indicate the probabilities of communication between the indicated pairs of ligands and receptors according to the color label. The strength of the interaction, as indicated by the presence of a dot and the color between ligands expressed on glia and cell surface receptors expressed on neurons in various clusters, are shown on the left. Similarly, cell surface receptors expressed on glia and ligands expressed on neurons in various clusters are shown on the right. The metadata underlying this figure can be found at https://zenodo.org/doi/10.5281/zenodo.10604562. (TIF) [file pbio.3002559.s014.tif]

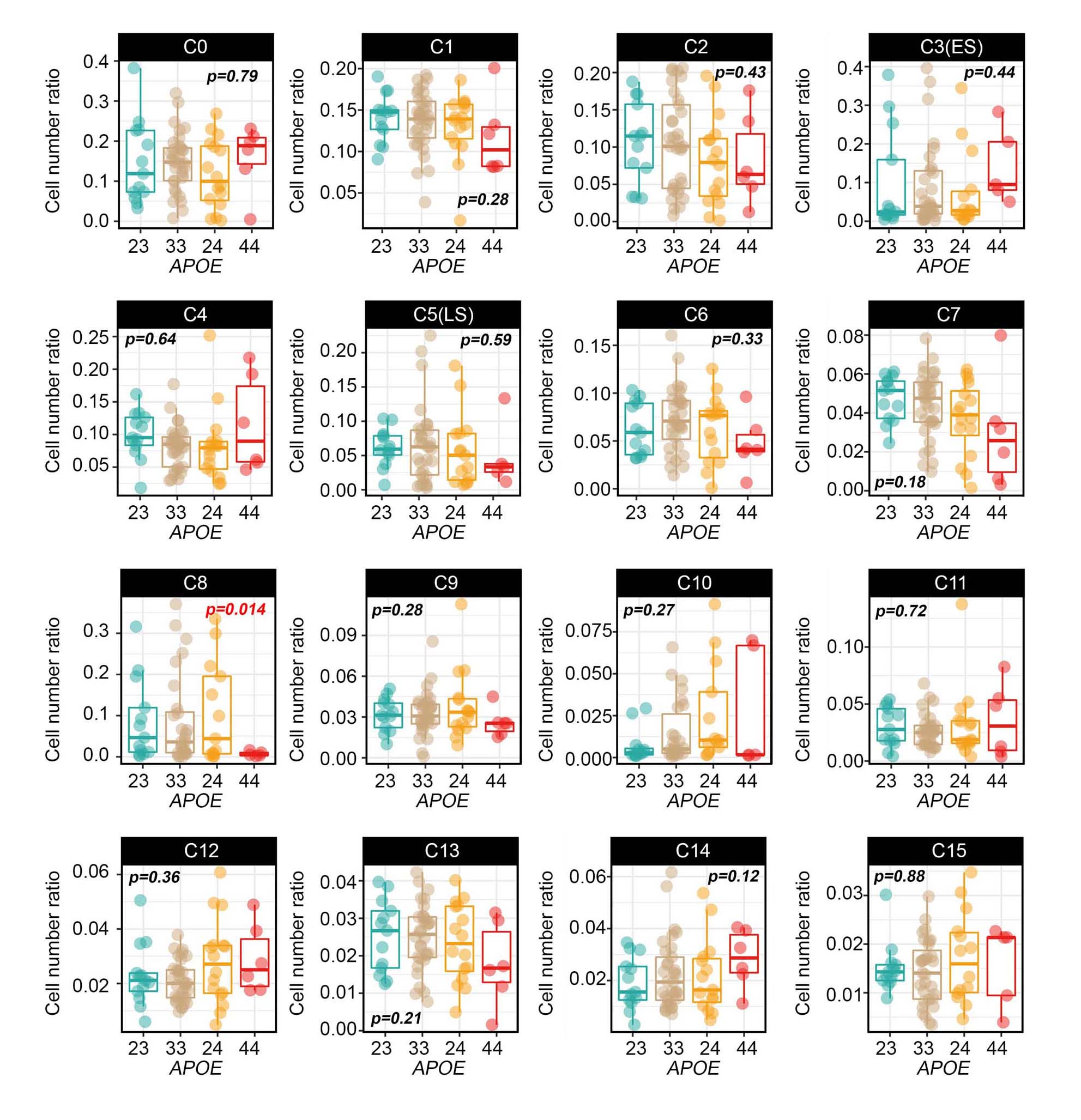

Supplement: S15 Fig — The metadata underlying this figure can be found at https://zenodo.org/doi/10.5281/zenodo.10604562. (JPG) [file pbio.3002559.s015.jpg]

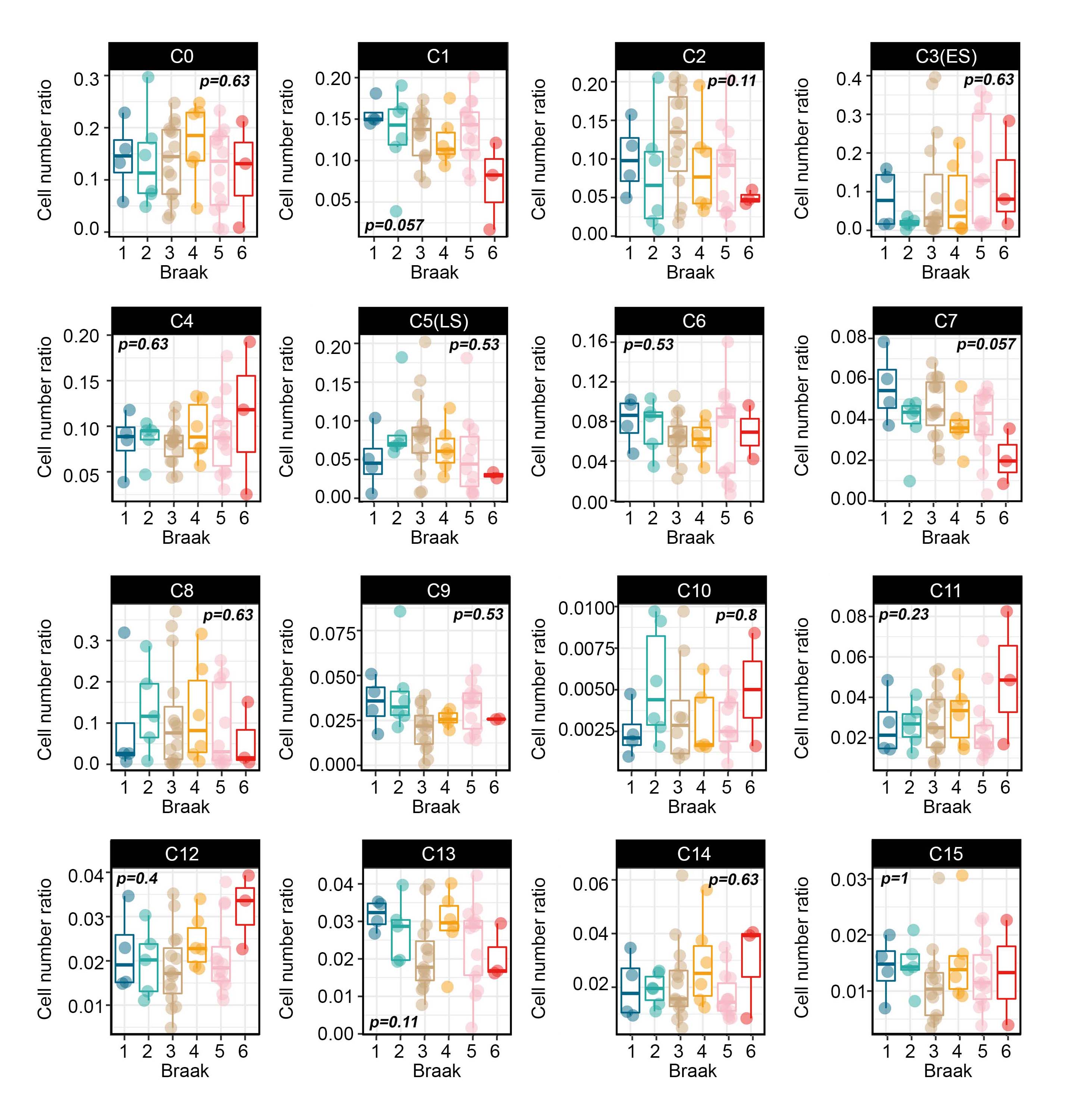

Supplement: S16 Fig — The metadata underlying this figure can be found at https://zenodo.org/doi/10.5281/zenodo.10604562. (JPG) [file pbio.3002559.s016.jpg]

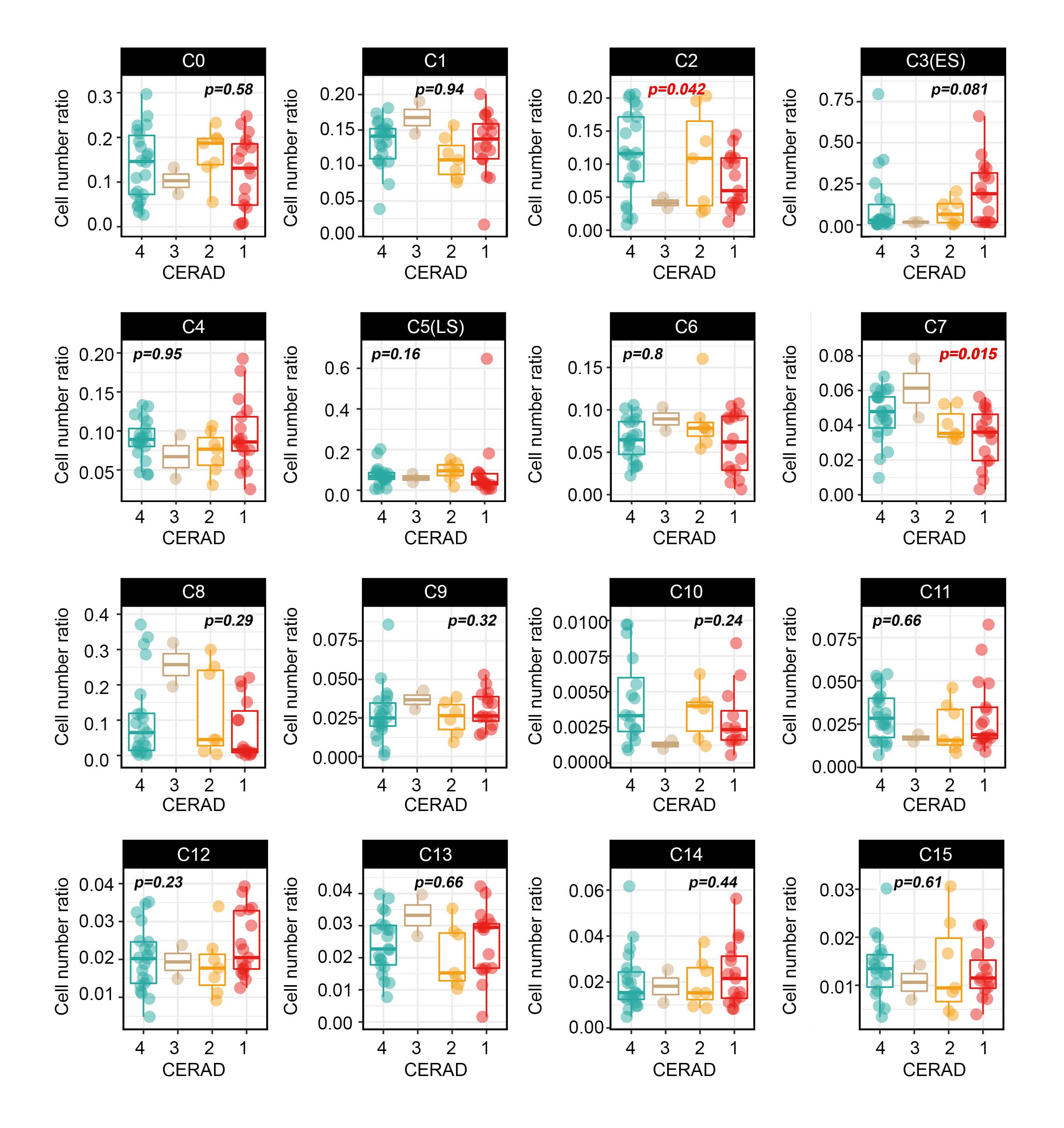

Supplement: S17 Fig — The metadata underlying this figure can be found at https://zenodo.org/doi/10.5281/zenodo.10604562. (JPG) [file pbio.3002559.s017.jpg]

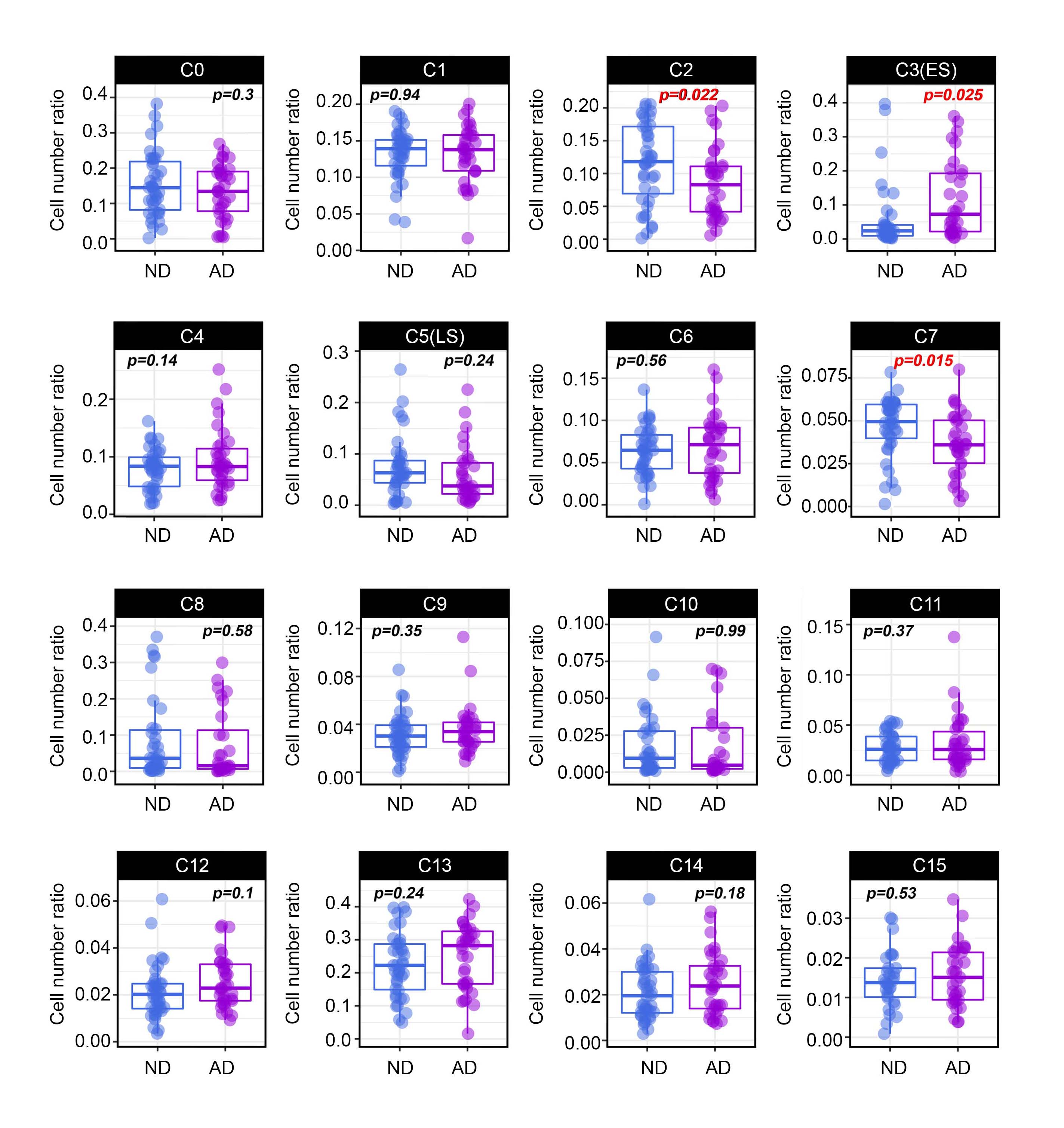

Supplement: S18 Fig — The metadata underlying this figure can be found at https://zenodo.org/doi/10.5281/zenodo.10604562. (JPG) [file pbio.3002559.s018.jpg]

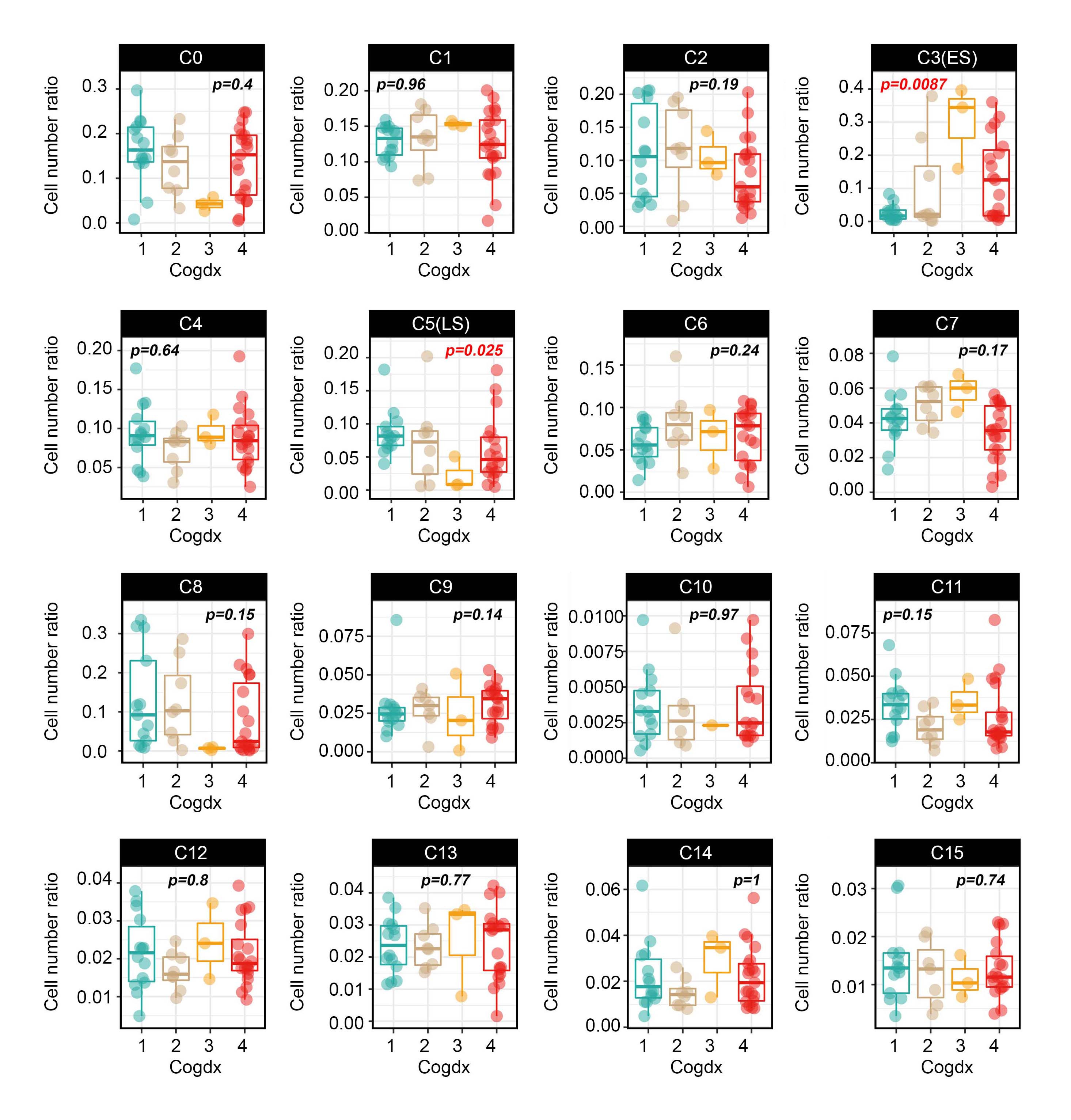

Supplement: S19 Fig — The metadata underlying this figure can be found at https://zenodo.org/doi/10.5281/zenodo.10604562. (JPG) [file pbio.3002559.s019.jpg]

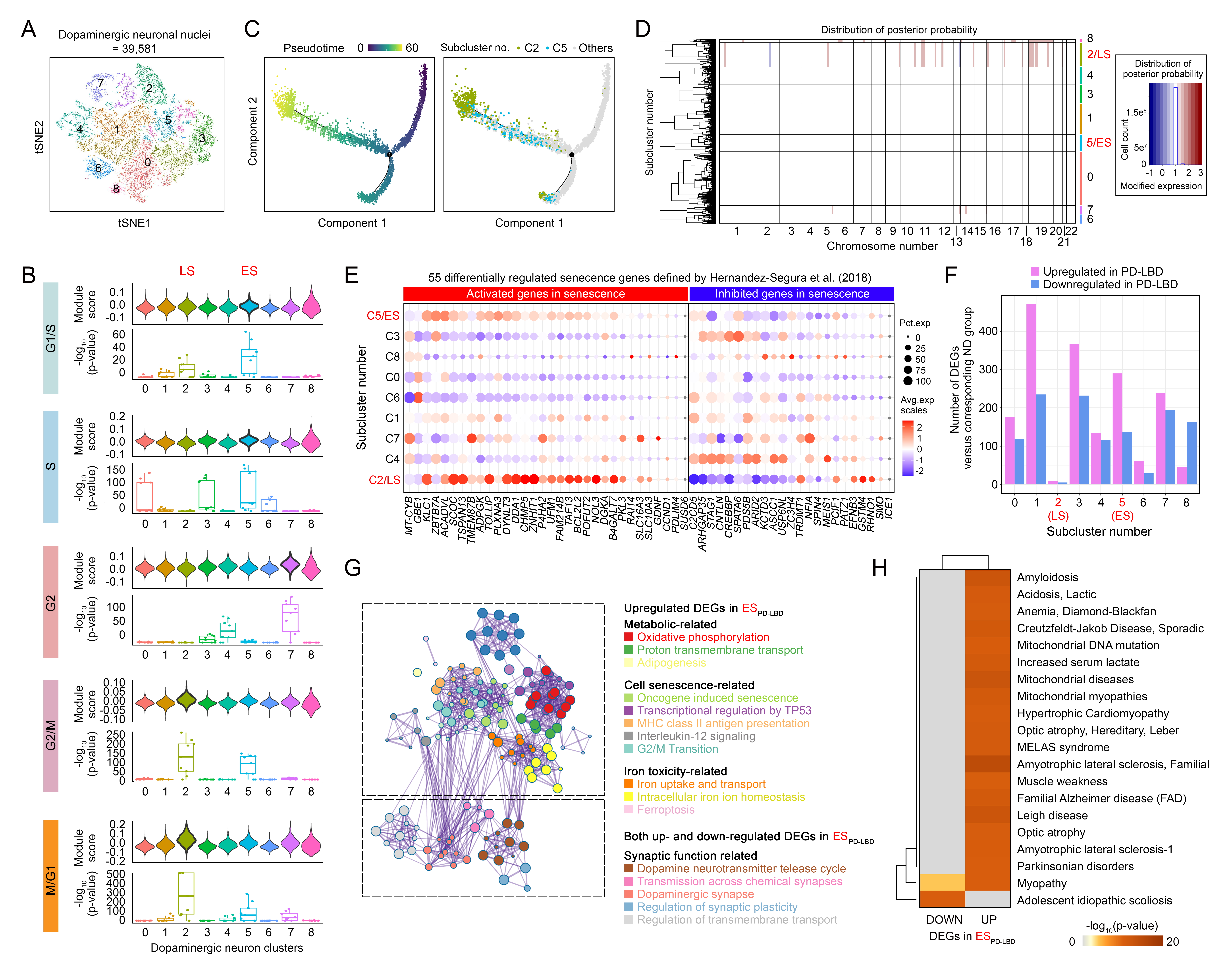

Supplement: S20 Fig — (A) t-SNE plot of dopaminergic neurons extracted from healthy and diseased mid-brain samples; these nuclei were divided into 9 subclusters (0–8). (B) Violin plots showing the distribution of cell cycle phase scores in all dopaminergic neuronal nuclei subclusters. The bold highlights indicate the subcluster with the most significant above-average gene expression levels in any particular cell cycle phase; p values against other subclusters are shown. (C) Single-cell trajectory analysis with the Monocle 2.0 algorithm revealing the evolutionary relationship between 2 subclusters of cell cycle gene-expressing neurons. Locations of subclusters 2 and 5 on this trajectory are labeled, indicating that they are on the same trajectory of fate, with subcluster 2 located at the terminal. (D) Estimation of copy number variants among all the dopaminergic neuronal nuclei extracted via the InferCNV algorithm. True positives of copy number variation events were identified in subcluster 2 (red: gain of copy number. Blue: loss of copy number). (E) Dot plot showing the expression levels of 55 differentially expressed senescence genes defined by Hernandez-Segura and colleagues [47] among all dopaminergic neuronal subclusters. (F) Bar plot representing the numbers of DEGs between groups of nuclei from PD-LBD patients and nondemented samples in all subclusters of dopaminergic neuronal nuclei. (G) Functional enrichment analysis of both up- and down-regulated DEGs in subcluster 5/ES (PD-LBD vs ND) was performed on the Metascape platform. (H) Bar plot showing the disease enrichment analysis of both up- and down-regulated DEGs in the ES cohort (PD-LBD vs ND) with the DisGenNet database. The metadata underlying this figure can be found at https://zenodo.org/doi/10.5281/zenodo.10604562. (TIF) [file pbio.3002559.s020.tif]

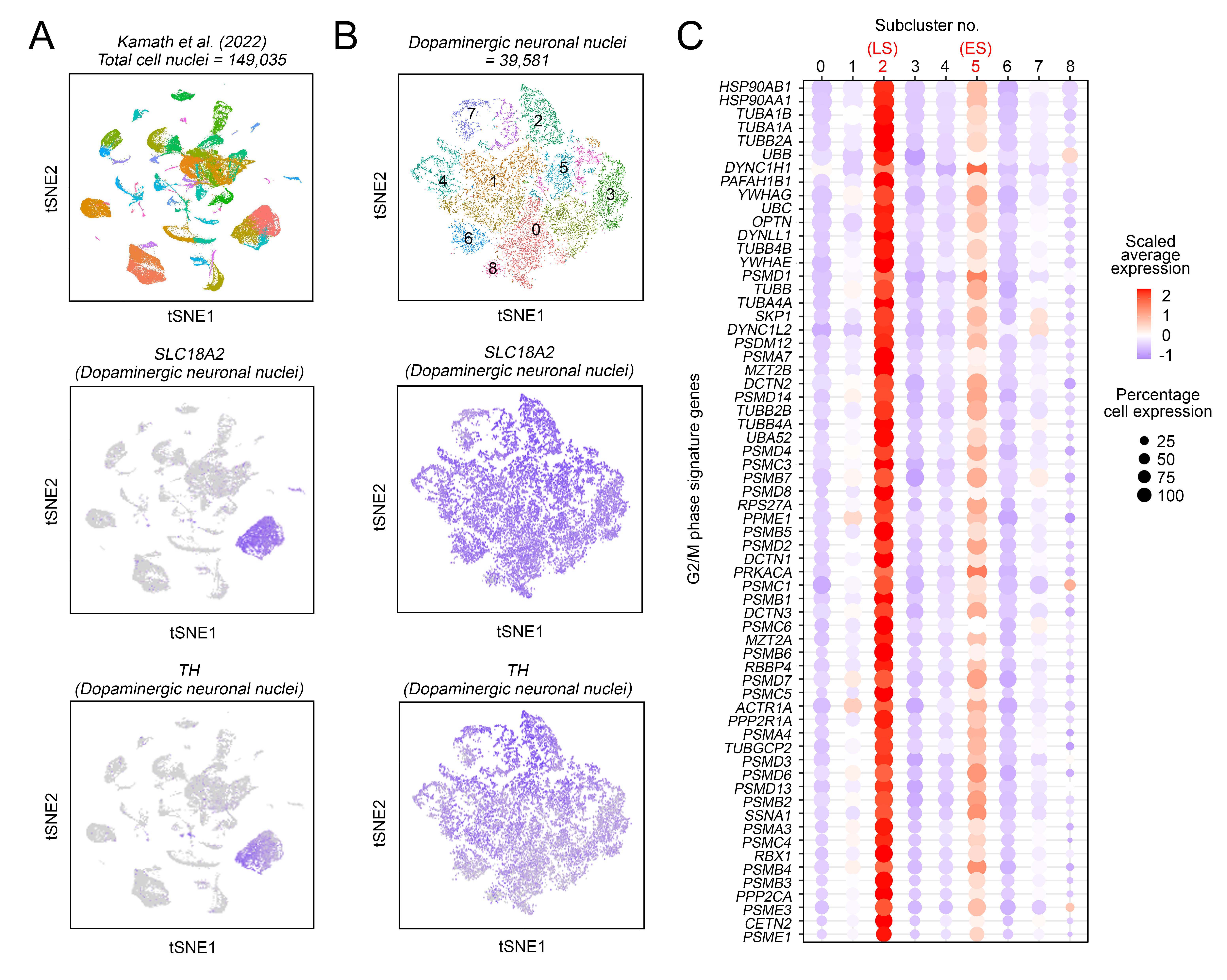

Supplement: S21 Fig — (A) t-SNE plots of all nuclei derived from both the unaffected and affected samples in the Kamath dataset [105]. Clusters are colored according to cell type identity. (B) Dopaminergic neurons were identified and selected based on 2 classic markers: SLC18A2 and TH. (C) Dot plot showing the scaled average expression levels of the classic G2/M genes among all the neuronal clusters. Elevated expression of most of these genes was identified in subclusters 5 (ES) and 2 (LS). The metadata underlying this figure can be found at https://zenodo.org/doi/10.5281/zenodo.10604562. (JPG) [file pbio.3002559.s021.jpg]

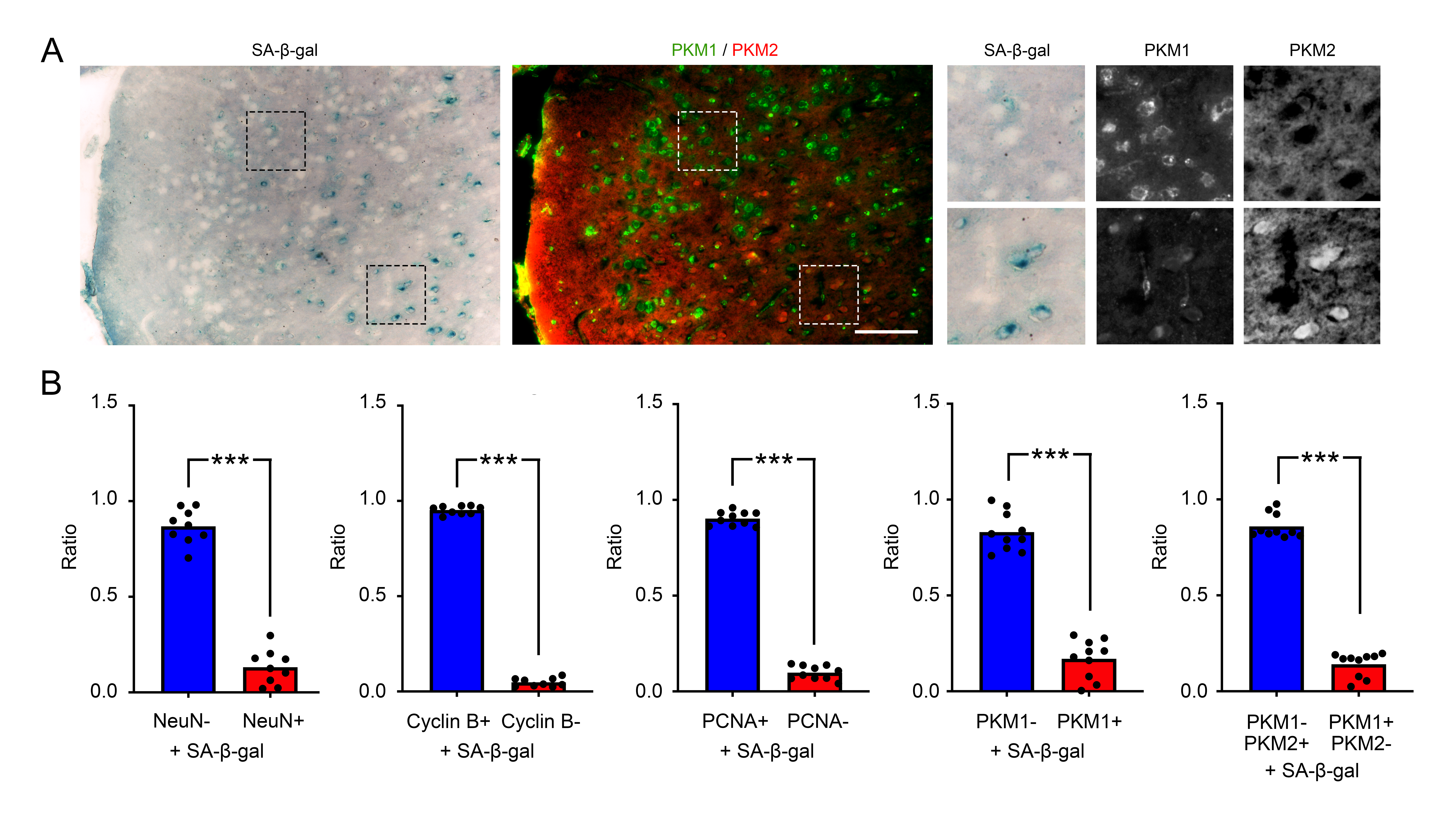

Supplement: S24 Fig — (A) Representative SA-β-gal and immunofluorescence staining images validating the inverse relationship between PKM1 and PKM2 expression in neurons [n = 10; quantification is shown in (B); scale bar = 200 μm]. (B) Quantification analyses of the brain histology data presented in S22L and S24A Figs (n = 10, ***p < 0.0001, unpaired t test). The metadata underlying this figure can be found at https://zenodo.org/doi/10.5281/zenodo.10604562. (TIF) [file pbio.3002559.s024.tif]
