# Supplementary material for: Neuronal cell cycle reentry events in the aging brain are more prevalent in neurodegeneration and lead to cellular senescence
Source: PLoS Biol. 2024 Apr 23;22(4):e3002559. doi: 10.1371/journal.pbio.3002559 (PMC11037540; doi:10.1371/journal.pbio.3002559)
Supplement: Attachment — Submitted filename: Point-to-point response.docx [file pbio.3002559.s035.docx]

**Point-to-point response**

**Reviewer #1:**

**Comment 1:** While the authors present results from an incredible amount of analyses, some of the conclusions are considered to be an over-interpretation of data. To confidently determine senescence and cell cycle re-entry, protein expression and post-translational modifications through histology is needed.

**Response 1:** Our team understand that the importance of confirming the findings with histology experiments and agree with the Reviewer’s suggestions. However, we do not have any frozen human brain samples available and the related ethics approval in the lab, nor there are brain banks that would allow the acquisition of post-mortem human AD brain samples easily in Hong Kong. On the other hand, while we also observed that the reviewer has offered an option of tuning down the conclusion if wet lab experiments cannot be included **(i.e., Reviewer 1, Comment 5),** we indeed communicated and agreed with the Senior Editor and Academic Editor on the option for providing additional mouse bioinformatics (with comparison to the human findings) and histology data to support the original findings.

Therefore, in response to the reviewer’s comment, the mouse related analyses are now added to the revised manuscript (**Revised manuscript: Fig.S22-23).** Since a mouse model that truly resembles late-onset AD does not exist and to the fact that aging is the primary risk factor for the disease [1], additional analyses were therefore conducted in a mouse model of aging (i.e., GSE207848, 21 month-old female C57BL/6J, frontal cortex) [2]. Similar to findings observed in human, a distinct sub-set of excitatory neurons **(Revised manuscript: Fig.S22B)** which exhibited no obvious cortical layer specific identity **(Revised manuscript: Fig.S22C-D)**, but showing induced cell cycle gene re-expression (**Revised manuscript: Fig.S22E)** as well as predicted signs of CNV **(Revised manuscript: Fig.S22F)** was identified (i.e., sub-cluster 2). Moreover, this sub-cluster of cells was also the only one group expressing the least number of total genes **(Revised manuscript: Fig.S22G)** and gene counts **(Revised manuscript: Fig.S22H),** while being the one with most robust expression of cell cycle genes **(Revised manuscript: Fig.S22E)** as relative to others. These observations indicated that they are more resembling the “late senescent (LS)” neurons defined in the human sample analyses. Subsequent comparisons in marker genes identified in LS neurons in mouse versus those from human (i.e., defined as Log2FC ≥ or ≤ 1; p < 0.01, as compared to the rest of the excitatory neuron populations) indicated substantial similarities between the two (i.e., 242 upregulated marker genes; 162 downregulated marker genes) **(Revised manuscript: Fig.S22I, S23B, Table.S9)**. Common downregulated LS marker genes were those implicated in neuronal specification and functions (i.e., synaptic activities and axonogenesis) **(Revised manuscript: Fig.S22K);** whereas common upregulated LS marker genes were those implicated in mitochondrial respiration and pyruvate metabolism **(Revised manuscript: Fig. S22J**). In the subsequent histology-based validation analyses, several marker genes were chosen from the common LS marker gene list **(Revised manuscript: Table.S9)** based on the existing knowledge on their roles in shaping the post-mitotic identity of neurons [3, 4], on the top of the classic markers that reflect cell cycle re-engagement and cellular senescence events occurring in neurons as published by our group previously [5, 6] **(Revised manuscript: Fig.S22L-N; S24)**. The rationales behind the chosen staining markers are elaborated as follow:

1. **Senescence-associated β-galactosidase (SA-β-gal) as the exploratory marker labelling senescent neurons**—This is a classic senescence marker which showed robust signals in senescent neurons in mouse brain sections, as reported by our team previously [5, 6] and indicated here by co-staining with NeuN **(Revised manuscript: Fig. S22L-N, S24).**
2. **Validation marker on the “cell cycle re-engaging” and “senescent” properties in neurons**—Signals of classic S-phase marker, such as nuclear signals of PCNA (i.e., Proliferating Cell Nuclear Antigen) and the global accumulation of G2/M-phase marker Cyclin B [7] were found in SA-β-gal-positive neurons. Moreover nuclear signals of Cyclin B could further indicate the trigger for permanent cell cycle exist and irreversible senescence response, and this may serve as an additional validation marker of neuronal senescence, as our team has reported previously [8] **(Revised manuscript: Fig. S22L, S22N, S24B).**
3. **Pyruvate kinase isozymes M1/M2 (PKM1/PKM2)**—The *PKM* gene was among one of those commonly upregulated LS marker genes despite the gene alternative splicing information (i.e., a cytosolic event) was not readily available from the single-nucleus RNA-transcriptomic data (i.e., detection of transcripts in nuclei). During neuronal maturation and differentiation, energy production shifts from glycolysis to oxidative phosphorylation, which is primarily driven by alternative splicing of pyruvate kinase M (PKM) pre-mRNA, switching from PKM2 to PKM1 production during brain development [3, 4]. PKM1 is the constitutively active tetrameric variant of the enzyme that functions only as the kinase of pyruvate in the cytoplasm; whereas the nature of PKM2 is more dynamic. When PKM2 exists in its tetrameric state, it functions similarly as PKM1. Once it dissociates into its dimeric/monomeric state, PKM2 may instead shuttle into the nucleus, where it moonlights and functions as a protein kinase instead [9]. In our immunohistology-based analyses, senescent neurons labelled by the robust SA-β-gal signal were positive for nuclear PKM2 signals; whereas their cytoplasmic signals of PKM1 were concurrently diminished **(Revised manuscript: Fig.S22M-N, S24).** In the contrary, their neighbouring non-senescent (i.e., SA-β-gal-negative) neurons revealed opposite patterns of these signals **(Revised manuscript: Fig.S22M-N, S24),** suggesting both the abundance and sub-cellular localization of PKM isozymes as a novel markers that distinguish senescent and non-senescent neurons.
4. **RNA binding Fox-1 Homolog3 (RBFOX3/NeuN)—**The *RBFOX3* gene was among one of those commonly downregulated LS markers, which encodes the neuronal nuclei (NeuN) antigen that has been widely used as a marker for post-mitotic neurons [10]. In both the human and mouse bioinformatics analyses, *RBFOX3* transcripts were lost uniquely in senescent neurons **(Response: Fig.1)** which the uniqueness of this trend of changes in senescent neurons was validated further by the immunohistology-based analyses **(Revised manuscript: Fig.S22L-M, S24B).** The loss in nuclear NeuN signals faithfully co-occurred in cells revealing positive signals of SA-β-gal, nuclear cyclin B and nuclear PKM2 signals **(Revised manuscript: Fig.S22L-M, S24B).**

Taken together, these cross-species observations not only validated the robustness of the findings from the human dataset analyses, but also opened up future opportunities to further study or modulate these cells in the laboratory mouse model setting.

**Response Fig.1.** tSNE plots of *RBFOX3/Rbfox3* gene expression level in the integrated human datasets (Left panel) and the mouse dataset (Right panel). Note that the cell cycling and senescent neurons (i.e., sub-clusters 3 and 5 in the integrated human datasets; sub-cluster 2 in the mouse dataset) consistently exhibited reduced expression of *RBFOX3/Rbfox3*.

**Comment 2:** A similar concern is noted regarding inferences about copy number variation and aneuploidy without confirmatory experiments. Moreover, canonical genes are missing from these processes; in order to confidently make conclusions from an incomplete "canonical signature", it is suggested to use independent bioinformatic approaches or test the hypothesis using biological analyses in cells or tissue.

**Response 2:** In response to the reviewer’s suggestions, we now supplemented the InferCNV data with a gene matching analysis, which would reveal if previously identified AD-related CNV gene loci [11] could be re-identified in the predicted inferCNV regions, following the correct trends of changes (i.e., CNV gain versus loss) **(Revised manuscript: Fig.4A-B, S13).** Similar to the findings observed in previous genome-wide or targeted gene analysis studies, CNV genes that were found to be lost in AD (e.g., *SDF4* at 1p36.33; *BIN1* at 2q14.3; *ERMP1* at 9p24; *VLDLR* at 9q24) could be matched to the predicted CNV regions in our analysis **(Revised manuscript: Fig.4A, S13A).** As previous study has suggested that CNV is linearly correlated with gene expression for the majority of genes [12], therefore the their gain or loss status of changes can be indirectly predicted from gene expression analysis with reference to negative controls. In both ES and LS neurons, expression of these CNV loss genes were consistently reduced, as compared to the rest of the excitatory neurons **(Revised manuscript: Fig.S13A, C)**. Likewise, CNV genes found to be gained in AD (e.g., *SLC30A3* at 2p23.3; *DNAJC5G* at 2p23.3, *TRIM54* at 2p23.3; *KLK6* at 19q13.3; *HAS* at 19q13.41 and *FPR1* at 19q13.41) could also be matched to the predicted CNV regions in our analyses as well **(Revised manuscript: Fig.4B).** Consistently, their expression levels in both ES and LS neurons were significant induced as well as compared to rest of other excitatory neurons **(Revised manuscript: Fig.S13B, C).** These matching findings on the “canonical signatures” shall therefore support the key findings in our subsequent analyses (**Revised manuscript: Fig.4A-B).**

**Comment 3:** The manuscript is missing some key references.

**Response 3:** We predict that this comment was mae in conjunction with the previous two comments related to both “neuronal cell cycle re-entry” and “CNVs in AD”. In the revised manuscript, additional references were included in both the introduction and results description parts accordingly.

**Comment 4:** Typos and a grammatical issues were noted throughout, but they can be resolved by the authors upon careful reading/editing.

**Response 4:** In response to the comment, we had consulted language editing services before the re-submission of the revised manuscript.

**Comment 5:** In summary, while the authors present an interesting data and hypotheses about their interpretations that merit publication, it is recommended that validations experiments are performed. Alternatively, the conclusions may be presented as suggestive inferences worth further exploration, instead of conclusive statements as provided here.

**Response 5:** In response to this comment and our justification mentioned above **(i.e., Response to Comment 1)**, we have tuned down the conclusions while at the same time added discussions on the limitations of the study, which highlighted that further validations in human samples will be necessary as a future direction in the revised manuscript.

**Reviewer #2:**

**Comment 1:** It is unclear how the criteria for determining aged, non-diseased brains and AD brains were established. It is important for the criteria to be consistent across the four cohort studies to ensure accurate comparisons. Additionally, providing a table summarizing the demographic information of the patients in each study would be highly beneficial for understanding the characteristics of the cohorts.

**Response 1:** In response to the reviewer’s suggestion, we have added a supplementary table **(Revised manuscript: Table.S1)** in the revised manuscript, where details of all samples included in our analysis were clearly elaborated. In brief, the non-demented samples from the Mathys et al. (2019) dataset [13] was used as the discovery cohort to identify the post-mitotic brain cell type(s) enriched with cell cycle gene re-expression and predicted InferCNVs. Then, in order to validate the observations derived from the discovery cohort, repeated analyses (i.e., in both integrated as well as separated analysis settings) with the Lau et al. (i.e., including ND and AD) [14], Yang et al. (i.e., only ND) [15], Nagy et al. (i.e., only ND) [16] and Mathys et al. [13] (i.e., AD) validation cohorts were conducted. In addition to these, extra sets of analyses were performed in a cohort of PD-LBD samples from the Kamath et al. study [17] and a set of mouse normal brain aging samples from Allen et al. [2].

**Comment 2:** The study employed multiple analytical approaches and various gene signatures including AD, cell cycle, and senescence, among others. However, the presentation of the results as a whole can be challenging for readers to comprehend. Therefore, the inclusion of a diagram illustrating the complete analytic procedure would greatly enhance the understanding of the study.

**Response 2:** We thank the reviewer’s suggestion. In the revised manuscript, we have included a schematic diagram **(Revised manuscript: Fig.1A)** which illustrated the work flow of our analytical strategy.

**Comment 3:** The analysis started with the snRNA-seq data from normal aging subjects in Mathys et al.'s study, followed by the inclusion of normal aging nRNA-seq data from three additional studies. Subsequently, the analysis was expanded to include the snRNA-seq data from AD patients in the same four studies. While conducting the analysis progressively is understandable, a more straightforward and potentially more effective approach would involve performing the clustering analysis of all the data from the four studies at the outset, followed by subsequent analyses from the perspectives of aging and AD/PD. This would lead to a unified clustering structure across different studies, simplifying comparisons and facilitating a comprehensive understanding of the findings.

**Response 3:** In response to the reviewer’s comment, we re-performed our analyses as instructed. In brief, after identifying excitatory neurons is the only post-mitotic cell type exhibiting signs of cell cycle re-entry from the discovery cohort **(Revised manuscript: Fig.1, S1-S2),** we performed a set of validation and target cell (i.e., excitatory neurons) characterization analyses with all the samples available from the fours studies in an integrated setting **(Revised manuscript: Fig.2-4, S3-S13)** prior subsequent comparative studies conducted between non-demented (ND) versus diseased-affected groups (i.e., AD or PD-LBD) **(Revised manuscript: Fig.4, S14-21).** In addition to this, we also included data obtained from separated investigations of each individual datasets in some of the key analyses, such as the InferCNV **(Revised manuscript: Fig.S5)**; cell fate trajectory analyses **(Revised manuscript: Fig.S7)**; SCENIC transcription regulon analysis **(Revised manuscript: Fig.S11)**, and AD risk gene loci analysis **(Revised manuscript: Fig.S12)**. These would help to demonstrate clearly that our conclusions drawn from the integrated analyses are robust and repeatable in each individual datasets, independent to the sample size effect.

**Comment 4:** A number of CNVs were identified, but their functional relevance was not well evaluated. It would be beneficial for the authors to investigate the association between CNVs and gene expression to assess their potential impact on cellular function.

**Response 4:** In response to this suggestion, we comprehensively evaluated the integrated functions of genes located in the predicted CNV gain and loss regions **(Revised manuscript: Fig.2G-H).** In brief, genes identified from the CNV gain regions were primarily related to mitogenic signalling pathways (i.e., PI3K/AKT/mTOR signalling, Wnt/β-catenin signalling, mTORC1 signalling); cell cycle events (i.e., Myc targets V1, Mitotic spindle); DNA damage response (i.e., DNA repair, UV Response Up, p53 pathway) and metabolic reprogramming (i.e., Oxidative phosphorylation, Adipogenesis, Cholesterol homeostasis) **(Revised manuscript: Fig.2G)**, versus those identified form predicted CNV-loss regions were more related to sex hormone response (i.e., Androgen response, Estrogen response early) and reactive oxygen species signalling **(Revised manuscript: Fig.2H).** These together hinted that the molecular properties of cell cycle re-engaging excitatory neurons were substantially different from the rest of the others. More importantly, we were also able to show that a significant portion of the marker genes (i.e., 32.6% of upregulated DEGs; 28.8% of downregulated DEGs) for late senescent (LS) neurons (i.e., defined Log2FC ≥ or ≤ 1; p < 0.01 against the rest of excitatory neurons) were indeed the same as the predicted CNV-localized genes **(Revised manuscript: Fig.S10A, C**). These were that indeed genes implicated in mitotic signalling **(Revised manuscript: Fig.S10B, Common UP)** and establishment of neuronal polarity and synaptic functions **(Revised manuscript: Fig.S10D, Common DOWN).**

**Reviewer #3:**

**Comment 1:** The data suggest non-randomly dysregulated regulated gene clusters at multiple chromosomal locations which is interpreted by the authors as arm-level aneuploidies. Did the authors also detect aneuploidies in these specific chromosome locations/arms by identifying loss of **genetic heterogeneity** in these genes compared to other genetic regions/cells that are not re-entering the cell-cycle?

**Response 1:** We thank the reviewer for the question. To be honest and frank, it is really difficult to validate the genetic heterogeneity predicted in our current analysis due to the limited sequencing read depths of the datasets (i.e., sequenced by 10X Genomics) and the lack of genomic mutation information. We did attempt to detect genetic heterogeneities by utilizing brain single nuclei transcriptomes profiled with SMART-Seq v4 RNA-sequencing, which offers full-length transcriptome analysis [18] (i.e., <https://portal.brain-map.org/atlases-and-data/rnaseq/human-multiple-cortical-areas-smart-seq>). However, transcriptomic data generated by this type of sequencing method rendered the detection of senescent neurons unfeasible, this is on one hand due to small number of total nuclei (i.e., 45,495 nuclei) being extracted across multiple human brain areas (i.e., average number of brain region-specific neuronal nuclei is small) , plus the dataset in concern used NeuN (i.e., *RBFOX3*) as a marker to sort out the neuronal nuclei, which would likely missed the ES and LS neuronal nuclei as they are ones deficient in *RBFOX3* **(Revised manuscript: Fig. S22L-M, S24, Table.S9) (Response: Fig.1).** Together, we believe SMART-Seq is not as good as 10X in studying this issue, due to the general small cell numbers in these datasets.

**Comment 2:** How well characterized is the expression of the 711 genes selected to represent the canonical cell-cycle pathways in the 10x chemistry? Is not clear whether all of these should be detected or the 357 reported are only expected to be detected given a lower expression of the remaining ones?

**Response 2:** We apologize for the unclear description in the old manuscript. As now elaborated in the revised version, the updated list indeed included a total of 1,231 transcripts, which constitutes 1,135 and 96 cell cycle-related transcripts curated by the Whitfield et al. [19] (i.e., covered mainly S and G2/M phase genes) and Tirosh et al. [20] (i.e., covered G1/S, S, G2, G2/M and M/G2 phase genes as well), respectively **(Revised manuscript: Table S2)**. Out of this collated gene list, a smaller list of 357 genes with official symbols which can be detected by 10x Genomics was refined. For the unconsidered transcripts were either ones annotated to hypothetical proteins, or those belonged expressed sequence tags (ESTs) or simply protein alias names which could be mapped to multiple official names. Moreover, as an initial quality control, gene candidates expressed in fewer than 3 cells were eliminated as well, such that the final list of 357 genes were at least expressed in 3 single cells **(Revised manuscript: Fig.1E, Table S2).**

**Comment 3:** How are the 711 cell-cycle genes detected in other single-cell technologies that provide deeper characterization of the transcriptome (for example smart-seq approaches).

**Response 3:** Linking this response to the details we described above **(Reviewer 3, Comment 2)**, we double checked and confirmed that 338 out of the refined list of 357 genes mentioned (Response: Table 1) could be detected in a SMART-seq dataset (i.e., <https://portal.brain-map.org/atlases-and-data/rnaseq/human-multiple-cortical-areas-smart-seq>) where samples were harvested from multiple brain regions. Moreover, in the SMART-seq setting, we adopted a more stringent cut off by considering the genes only if they are expressed in more than 0.1% nuclei. As a result, a list of 338 genes were refined in the SMART-seq whereas 357 genes could be considered in the 10X Genomics setting. This outcome suggested that the cell cycle gene list utilized in our analyses was not dependent on the sequencing platform, but instead it is a cellular property of various brain cells.

**Comment 4:** The data reported in line 99 indicated a mean of ~1,400 nucleus per brain, which is considerably low for nowadays standards. Do the authors detected similar transcriptomic signatures and proportions in more recently generated datasets using latest 10x chemistries?

**Response 4:** We agree with the reviewer that the relatively low number of nuclei detected in each sample of the Mathys et al. dataset [13] may potentially affect the findings and conclusions. Therefore, in our manuscript, we also conducted separated analyses in three independent datasets, including the Lau et al. (8,346 nuclei/brain) [14], Nagy et al. (2,071 nuclei/brain) [16] and the Yang et al. (3,446 nuclei/brain) [15] data **(Revised manuscript: Fig.S3-S5, S7, S11-S12**). Consistent findings and conclusion could be drawn from these independent analyses, thus indicated our findings are robust.

**Comment 5:** Additional details about the subcluster and further characterization should be provided: What are the neuronal characteristics of cells in clusters 5 and 13? How are thse compared to other neuronal subclusters? Are these reflecting cortical layers?

**Response 5:** In response to this comment, we now comprehensively elaborated these details based on the updated integrated analyses setting of the 4 datasets (i.e., Sub-cluster 5_Mathys et al._ = Sub-cluster 3_Integrated_ ; Sub-cluster 13_Mathys et al._ = Sub-cluster 5_Integrated_).

In the revised manuscript, we illustrated that Sub-cluster 3_Integrated_ was likely derived from layers II-III despite the expression levels of key layer-specific markers were substantially diminished **(Revised manuscript: Fig.3A-B, S6)**. For the Sub-cluster 5_Integrated_, these cells however had almost lost all their cortical layer information, and was predicted to behave distinctively form all other excitatory neurons **(Revised manuscript: Fig.3A-B, S6)**. Therefore, additional characterization of these cells in comparison to others, such as the functional anlayses of genes enriched in the predicted CNV gain and loss regions **(Revised manuscript: Fig2G)**, cell cycle genes detected **(Revised manuscript: Fig.3F)** and specific AD risk genes enriched **(Revised manuscript: Fig.4A-B, S12-13)** were performed. Moreover, detailed analyses of activated SCENIC transcriptomic regulons **(Revised manuscript: Fig.3I, S11),** so as ligand-receptor-mediated communication with glia **(Revised manuscript: Fig.4E, S14)** were also conducted and added in the revised manuscript.

**Comment 6:** Actual p-values should be provided, for example in line 149.

**Response 6:** In response to this comment, the p-value of the CNV comparison between Sub-cluster 13 versus others was added **(Revised manuscript: Line 158)**, and whenever applicable, actual p-values were either directly labelled in the figures or included supplementary tables.

**Comment 7:** As currently phrased, it seems that data from Mathys et al (ref 11) is used both in discovery (line 98) and replication (line 163). What are the demographics, in particular the age at death, of the data used in additional cohorts? Additional datasets for AD/PD brain cohorts are available, but instead the authors selected tissue from other traits/diseases without explanation.

**Comment 7:** We apologize for the incorrect description in the old manuscript, and here we clarify that only the non-demented (ND) samples from the Mathys et al. dataset [13] was used as a discovery cohort **(Revised manuscript: Line 103)**, and subsequently the AD samples of this dataset (i.e., Mathys et al.) [13] along with the Lau et al. [14], Nagy et al. [16] and the Yang et al. datasets were used as the validation cohorts in this study. These dataset details were now elaborated in Table.S1 of the revised manuscript.

Indeed, we did consider other brain sample cohorts, such as the dataset from Grubman et al. [21] and Morabito et al. [22] in our analysis despite they were not shown in the manuscript due to the following reasons. For the analyses with the Grubman et al. dataset [21], the samples were obtained from the entorhinal cortex where indeed this brain region constitutes only limited number of excitatory neurons [i.e., 656 excitatory neuronal nuclei / 13,214 total number of nuclei (4.96%)] and was therefore not considered in the subsequent analyses. For the analyses with the Morabito et al. dataset [22], since the study was focusing on oligodendrocytes **(Response: Fig.2A-D)** and again very limited number of excitatory neurons could be extracted [i.e., 3,299 excitatory neuronal nuclei / 57,153 total number of nuclei (5.77%)], therefore this dataset was not integrated into the analyses elaborated in our manuscript.

Nevertheless, here we would like to illustrate to the reviewers that even with such a limitations in the Morabito et al. dataset [22], we were still able to identify a sub-population of excitatory neurons that re-expressed cell cycle genes, showing positive signs of predicted CNV signals, loss in cortical layer markers and heightened expression of senescent genes **(Response: Fig.2)**.

**Response Fig. 2.** Identification and characterization of cell cycle re-engaging and senescent neurons in the Morabito et al. dataset [22]. **A**. UMAP plot of the total 57,153 sequenced nuclei from the prefrontal cortex. Colour code represented different cell types: *excitatory neurons, Ex; oligodendrocytes, Oli; inhibitory neurons, In; astrocytes, Ast; microglia, Mic; endothelial cells, End; Oligodendrocyte progenitor cells, Opc; Fibroblast, Fib; T cells, Tcell*. The histogram at the bottom illustrates the statistics of nuclei number in different cell types. **B.** Expression patterns of neuronal-associated markers which helps to define the excitatory (i.e., *NRGN*) and inhibitory (i*.e., GAD2*) neurons. C. Violin plots displaying the expression levels of various cell type specific markers. **D**. Left: UMAP plot of the 3,470 excitatory neuronal nuclei. Colour code represents different sub-clusters. Right: UMAP plot illustrate the statistics of total expressed gene numbers in different neuronal sub-clusters. **E**. Violin plots showing cell cycle phase scores of all sub-clusters of excitatory neuronal nuclei. Bold violin highlights the sub-clusters with most significant above average gene expression levels among all sub-clusters in the same cell cycle phase. The corresponding significance values of each violin against the rest are shown. **F.** Estimation of copy number variants by the InferCNV algorithm in all sub-clusters of excitatory neurons. The heatmap shows the copy number variation regions identified by the Hidden Markov Model [i.e., regions of gain (red) and loss (blue) in expression along each chromosome from the p-arm (left side of each box) to the q-arm (right side of each box)] in all sub-clusters. **G.** Dot plot presents the expression levels of cerebral cortex layer-specific markers among all excitatory neuronal sub-clusters. **H.** Single-cell trajectory analysis with the Monocle algorithm identifies an evolutionary relationship between the two cell cycle gene re-expressing neuronal sub-clusters 5 and 6, from which nuclei of sub-cluster 5 are more enriched at the terminal of the branch. **I.** Dot plot presents the expression levels of the 55 senescence genes identified by Hernandez-Segura et al. [23] in all sub-types of excitatory neurons.

**Comment 8:** Datasets should be described properly.

**Response 8:** In the revised manuscript, a supplementary table **(Revised manuscript: Table S1)** was added to describe all sample details from each dataset utilized in the manuscript.

**Comment 9:** Specific pipelines to integrate and single-cell data are available, but not employed by the authors. Similarly, there are methods proposed in the literature to evaluate the accuracy of the integration that should be employed by the authors.

**Response 9:** We agreed with the reviewer that different integration methodologies and analytical parameters could substantially impact on the cell sub-cluster identification, leading to false discoveries. Therefore in the revised manuscript, we conducted a stringent quality control analysis to validate that the identification of cell cycle re-engaging and senescent neurons was not dependent on the analytical approach. Firstly, Harmony [24] the alternative integration method was used to integrate the datasets, from which a consistent cluster distribution was observed with that resulted from the rPCA method **(Revised manuscript: Fig. S3C-H).** On the top of that, we also tested a wide range of resolutions to quantify their potential impact on sub-cluster identification. It is noticeable that the senescent neuronal cluster could further be divided into smaller sub-clusters as higher the resolution value was set. Despite so, none of these cells rendered disoriented and became mixed with any non-cell cycle re-engaging neurons **(Revised manuscript: Fig.S3A-B)**. This observation therefore indicated that the transcriptomic signature of cell cycle re-engaging and senescent neurons are unique from the rest of the excitatory neuronal population.

**Comment 10:** The authors keep clustering the cells for distinct analyses which makes the paper difficult to follow un understand. Although I fully support subclustering of cells types, it is in my opinion that it should be done only once and conserved on the entire paper. Clustering processes are highly sensitive to critical parameters, and might produce divergent results that should be critically analyzed otherwise.

**Response 10:** We appreciate the reviewer’s suggestions. Therefore in the revised manuscript, we integrated all datasets in concern at the outset **(Revised manuscript: Fig.2, S4-5)** right after identifying that excitatory neuron is the only post-mitotic cell type that contained sub-populations of cell nuclei with enriched expression of cell cycle genes and predicted signs of arm- or chromosomal aneuploidy **(Revised manuscript: Fig.1, S1-2)**. Based on this updated setting, the same sub-cluster number was adopted to the rest of the entire work related to AD **(Revised manuscript: Fig.3-4, S3-S19)**. The cell cycle re-engaging and senescent neurons identified in this current setting [i.e., including both non-demented (ND) and AD samples] revealed a consistent phenotypic signatures as compared to those described in the original manuscript (i.e., that based on ND samples), these included similar patterns of predicted inferCNV profiles **(Revised manuscript: Fig.2E, S5)**, the existence of early and late stage of senescence in the cell trajectory analysis **(Revised manuscript: Fig.3D, S7)**; their gradual loss in cortical layer identifies (i.e., from early to late senescent stages) **(Revised manuscript: Fig.3A-B, S6)**, and the emergence of commonly known cellular senescence-associated signatures at late stage **(Revised manuscript: Fig.3G).**

**Comment 11:** How coincident are the (sub)clusters identified to the ones reported previously?

**Response 11:** Indeed, this rare cell cycle re-engaging and senescent neuronal sub-cluster were identified in previous studies despite they were either not being clearly defined or even being discarded in the subsequent analysis in the previous literatures [25, 26]. We are confident that the senescent neuronal cluster was indeed characterized by the abnormally heightened expression level of *NRGN* as compared to the rest of the excitatory neuronal population **(Response: Fig.3).** According to the Velmeshev D et al. study, they also identified a similar cluster which was named as the *NRGN-*expressing neurons and the authors also revealed that they did not express any cortical layer markers [25]. Similarly in the recent Mathys et al. paper, they also revealed the presence of *NRGN*-enriched sub-cluster called “Exc NRGN” despite they had excluded this sub-cluster in the rest of their analysis [26].

**Response Fig. 3.** tSNE plots of *NRGN//Nrgn* gene expression level in the integrated human datasets (Left panel) and the mouse dataset (Right panel). Note that the cell cycling neurons (i.e., sub-clusters 3 and 5 in the integrated human datasets; sub-cluster 2 in the mouse dataset) consistently exhibited reduced expression of *NRGN/Nrgn*.

**Comment 12**: Although methods to integrate datasets are evolving rapidly, it is still considered a best practive to analyze each dataset by itself, instead of the integrated data from multiple cohorts/brain regiosn, etc. Otherwise, spurious results can be incorporated by any technical variable/artifact proper of any datasets. This affect regulatory network analyses (SCENIC) that should be done for each datasets and then combined.

**Response 12:** We agree with the reviewer’s viewpoint that the gene quantifications shall be done separately to avoid any potential artefacts. Therefore in the revised manuscript, the SCENIC analyses for each individual dataset was included **(Revised manuscript: Fig.S11, Table.S7)** which indeed indicated that the activated and inhibited transcription regulons predicted from each datasets were highly similar. Moreover, we also extended this in other analyses, including the InferCNV (**Revised manuscript: Fig. S5)** and pseudo-time trajectory **(Revised manuscript: Fig.S7)** analyses. Both of them revealed consistent results against those obtained from the integrated dataset setting. These indicated that the conclusions we drawn from the integrated outset were robust and valid.

**Comment 13:** The methods lack details (for example the differential gene expression analyses).

**Response 13:** In response to this comment, we have updated the methods section in the revised manuscript. Specifically, for differentially expressed gene (DEG) expression analysis, we updated the text by adding “The FindMarker function in the Seurat package was used with the Mast test having the dataset source, sample sex and sample age set as covariates. The marker genes for LS neurons were considered only when p-value is smaller than <0.01 and Abs(log2FC)>1. The DEGs between ES neurons of AD versus ND groups were only considered when the p-value is < 0.01.

**References list:**

1.	Hou Y, Dan X, Babbar M, Wei Y, Hasselbalch SG, Croteau DL, et al. Ageing as a risk factor for neurodegenerative disease. Nat Rev Neurol. 2019;15(10):565-81. Epub 2019/09/11. doi: 10.1038/s41582-019-0244-7. PubMed PMID: 31501588.

2.	Allen WE, Blosser TR, Sullivan ZA, Dulac C, Zhuang X. Molecular and spatial signatures of mouse brain aging at single-cell resolution. Cell. 2023;186(1):194-208 e18. Epub 2022/12/30. doi: 10.1016/j.cell.2022.12.010. PubMed PMID: 36580914; PubMed Central PMCID: PMCPMC10024607.

3.	Zheng X, Boyer L, Jin M, Mertens J, Kim Y, Ma L, et al. Metabolic reprogramming during neuronal differentiation from aerobic glycolysis to neuronal oxidative phosphorylation. Elife. 2016;5. Epub 2016/06/11. doi: 10.7554/eLife.13374. PubMed PMID: 27282387; PubMed Central PMCID: PMCPMC4963198.

4.	Su CH, Hung KY, Hung SC, Tarn WY. RBM4 Regulates Neuronal Differentiation of Mesenchymal Stem Cells by Modulating Alternative Splicing of Pyruvate Kinase M. Mol Cell Biol. 2017;37(3). Epub 2016/11/09. doi: 10.1128/MCB.00466-16. PubMed PMID: 27821480; PubMed Central PMCID: PMCPMC5247614.

5.	Sun JK, Wu D, Wong GC, Lau TM, Yang M, Hart RP, et al. Chronic alcohol metabolism results in DNA repair infidelity and cell cycle-induced senescence in neurons. Aging Cell. 2023;22(2):e13772. Epub 2023/01/24. doi: 10.1111/acel.13772. PubMed PMID: 36691110; PubMed Central PMCID: PMCPMC9924945.

6.	Chow HM, Shi M, Cheng A, Gao Y, Chen G, Song X, et al. Age-related hyperinsulinemia leads to insulin resistance in neurons and cell-cycle-induced senescence. Nat Neurosci. 2019;22(11):1806-19. Epub 2019/10/23. doi: 10.1038/s41593-019-0505-1. PubMed PMID: 31636448.

7.	Lara-Gonzalez P, Moyle MW, Budrewicz J, Mendoza-Lopez J, Oegema K, Desai A. The G2-to-M Transition Is Ensured by a Dual Mechanism that Protects Cyclin B from Degradation by Cdc20-Activated APC/C. Dev Cell. 2019;51(3):313-25 e10. Epub 2019/10/08. doi: 10.1016/j.devcel.2019.09.005. PubMed PMID: 31588029; PubMed Central PMCID: PMCPMC7778526.

8.	Charrier-Savournin FB, Chateau MT, Gire V, Sedivy J, Piette J, Dulic V. p21-Mediated nuclear retention of cyclin B1-Cdk1 in response to genotoxic stress. Mol Biol Cell. 2004;15(9):3965-76. Epub 2004/06/08. doi: 10.1091/mbc.e03-12-0871. PubMed PMID: 15181148; PubMed Central PMCID: PMCPMC515331.

9.	Zhang Z, Deng X, Liu Y, Liu Y, Sun L, Chen F. PKM2, function and expression and regulation. Cell Biosci. 2019;9:52. Epub 2019/08/09. doi: 10.1186/s13578-019-0317-8. PubMed PMID: 31391918; PubMed Central PMCID: PMCPMC6595688.

10.	Gusel'nikova VV, Korzhevskiy DE. NeuN As a Neuronal Nuclear Antigen and Neuron Differentiation Marker. Acta Naturae. 2015;7(2):42-7. Epub 2015/06/19. PubMed PMID: 26085943; PubMed Central PMCID: PMCPMC4463411.

11.	Cuccaro D, De Marco EV, Cittadella R, Cavallaro S. Copy Number Variants in Alzheimer's Disease. J Alzheimers Dis. 2017;55(1):37-52. Epub 2016/11/02. doi: 10.3233/JAD-160469. PubMed PMID: 27662298; PubMed Central PMCID: PMCPMC5115612.

12.	Shao X, Lv N, Liao J, Long J, Xue R, Ai N, et al. Copy number variation is highly correlated with differential gene expression: a pan-cancer study. BMC Med Genet. 2019;20(1):175. Epub 2019/11/11. doi: 10.1186/s12881-019-0909-5. PubMed PMID: 31706287; PubMed Central PMCID: PMCPMC6842483.

13.	Mathys H, Davila-Velderrain J, Peng Z, Gao F, Mohammadi S, Young JZ, et al. Single-cell transcriptomic analysis of Alzheimer's disease. Nature. 2019;570(7761):332-7. Epub 2019/05/03. doi: 10.1038/s41586-019-1195-2. PubMed PMID: 31042697; PubMed Central PMCID: PMCPMC6865822.

14.	Lau SF, Cao H, Fu AKY, Ip NY. Single-nucleus transcriptome analysis reveals dysregulation of angiogenic endothelial cells and neuroprotective glia in Alzheimer's disease. Proc Natl Acad Sci U S A. 2020;117(41):25800-9. Epub 2020/09/30. doi: 10.1073/pnas.2008762117. PubMed PMID: 32989152; PubMed Central PMCID: PMCPMC7568283.

15.	Yang AC, Kern F, Losada PM, Agam MR, Maat CA, Schmartz GP, et al. Dysregulation of brain and choroid plexus cell types in severe COVID-19. Nature. 2021;595(7868):565-71. Epub 2021/06/22. doi: 10.1038/s41586-021-03710-0. PubMed PMID: 34153974; PubMed Central PMCID: PMCPMC8400927.

16.	Nagy C, Maitra M, Tanti A, Suderman M, Theroux JF, Davoli MA, et al. Single-nucleus transcriptomics of the prefrontal cortex in major depressive disorder implicates oligodendrocyte precursor cells and excitatory neurons. Nat Neurosci. 2020;23(6):771-81. Epub 2020/04/29. doi: 10.1038/s41593-020-0621-y. PubMed PMID: 32341540.

17.	Kamath T, Abdulraouf A, Burris SJ, Langlieb J, Gazestani V, Nadaf NM, et al. Single-cell genomic profiling of human dopamine neurons identifies a population that selectively degenerates in Parkinson's disease. Nat Neurosci. 2022;25(5):588-95. Epub 2022/05/06. doi: 10.1038/s41593-022-01061-1. PubMed PMID: 35513515; PubMed Central PMCID: PMCPMC9076534 competing interests.

18.	Ramskold D, Luo S, Wang YC, Li R, Deng Q, Faridani OR, et al. Full-length mRNA-Seq from single-cell levels of RNA and individual circulating tumor cells. Nat Biotechnol. 2012;30(8):777-82. Epub 2012/07/24. doi: 10.1038/nbt.2282. PubMed PMID: 22820318; PubMed Central PMCID: PMCPMC3467340.

19.	Whitfield ML, Sherlock G, Saldanha AJ, Murray JI, Ball CA, Alexander KE, et al. Identification of genes periodically expressed in the human cell cycle and their expression in tumors. Mol Biol Cell. 2002;13(6):1977-2000. Epub 2002/06/12. doi: 10.1091/mbc.02-02-0030. PubMed PMID: 12058064; PubMed Central PMCID: PMCPMC117619.

20.	Tirosh I, Izar B, Prakadan SM, Wadsworth MH, 2nd, Treacy D, Trombetta JJ, et al. Dissecting the multicellular ecosystem of metastatic melanoma by single-cell RNA-seq. Science. 2016;352(6282):189-96. Epub 2016/04/29. doi: 10.1126/science.aad0501. PubMed PMID: 27124452; PubMed Central PMCID: PMCPMC4944528.

21.	Grubman A, Chew G, Ouyang JF, Sun G, Choo XY, McLean C, et al. A single-cell atlas of entorhinal cortex from individuals with Alzheimer's disease reveals cell-type-specific gene expression regulation. Nat Neurosci. 2019;22(12):2087-97. Epub 2019/11/27. doi: 10.1038/s41593-019-0539-4. PubMed PMID: 31768052.

22.	Morabito S, Miyoshi E, Michael N, Shahin S, Martini AC, Head E, et al. Single-nucleus chromatin accessibility and transcriptomic characterization of Alzheimer's disease. Nat Genet. 2021;53(8):1143-55. Epub 2021/07/10. doi: 10.1038/s41588-021-00894-z. PubMed PMID: 34239132; PubMed Central PMCID: PMCPMC8766217.

23.	Hernandez-Segura A, de Jong TV, Melov S, Guryev V, Campisi J, Demaria M. Unmasking Transcriptional Heterogeneity in Senescent Cells. Curr Biol. 2017;27(17):2652-60 e4. Epub 2017/08/29. doi: 10.1016/j.cub.2017.07.033. PubMed PMID: 28844647; PubMed Central PMCID: PMCPMC5788810.

24.	Korsunsky I, Millard N, Fan J, Slowikowski K, Zhang F, Wei K, et al. Fast, sensitive and accurate integration of single-cell data with Harmony. Nat Methods. 2019;16(12):1289-96. Epub 2019/11/20. doi: 10.1038/s41592-019-0619-0. PubMed PMID: 31740819; PubMed Central PMCID: PMCPMC6884693.

25.	Velmeshev D, Schirmer L, Jung D, Haeussler M, Perez Y, Mayer S, et al. Single-cell genomics identifies cell type-specific molecular changes in autism. Science. 2019;364(6441):685-9. Epub 2019/05/18. doi: 10.1126/science.aav8130. PubMed PMID: 31097668; PubMed Central PMCID: PMCPMC7678724.

26.	Mathys H, Peng Z, Boix CA, Victor MB, Leary N, Babu S, et al. Single-cell atlas reveals correlates of high cognitive function, dementia, and resilience to Alzheimer's disease pathology. Cell. 2023;186(20):4365-85 e27. Epub 2023/09/30. doi: 10.1016/j.cell.2023.08.039. PubMed PMID: 37774677; PubMed Central PMCID: PMCPMC10601493.
