# Supplementary material for: Neuronal cell cycle reentry events in the aging brain are more prevalent in neurodegeneration and lead to cellular senescence
Source: PLoS Biol. 2024 Apr 23;22(4):e3002559. doi: 10.1371/journal.pbio.3002559 (PMC11037540; doi:10.1371/journal.pbio.3002559)
Supplement: Attachment — Submitted filename: Point-to-point response.docx [file pbio.3002559.s037.docx]

**Point-to-point response**

**Response to the editorial requests:**
**Comment 1**—TITLE: After some discussion within the team, we think the title could be strengthened if it conveyed more of the specific findings of the study. If you agree (and if supported), we suggest you change it to something like: "Neuronal cell cycle re-entry events in the aging brain are more prevalent in neurodegeneration and lead to cellular senescence"

**Response 1**—We agree with the editorial requests and amended the title as suggested.

**Comment 2**—ETHICS STATEMENT: Please update the ethics statement in your manuscript to provide the approval number for the protocol, approved by the ethics committee of CUHK.

**Response 2**—In the revised manuscript, the approval number (19/243/GRF) is added back to the ethics statement.

**Comment 3**—DATA/CODE: Thank you for providing the data and code underlying your manuscript on Github. Can you please generate a DOI for this dataset and code? This can be done with zenodo. <https://docs.github.com/en/repositories/archiving-a-github-repository/referencing-and-citing-content>

**Response 3**—In the revised manuscript, we followed the editor’s instructions and replaced the original link with the following link generated by Zenodo: <https://zenodo.org/doi/10.5281/zenodo.10604562> .

**Comment 4**—Can you please also add a brief sentence to each relevant figure legend referencing pointing readers to this repository. For example , you can add the sentence "the data underlying this figure can be found at ___"

**Response 4**—In the revised manuscript, we followed the editor’s instructions and added the following statement “The metadata underlying this figure can be found at <https://zenodo.org/doi/10.5281/zenodo.10604562>.” in Figure the legends of Figures 1, 2, 3, 4, S2, S3, S4, S5, S8, S9, S10, S11, S12, S13, S14, S15, S16, S17, S18, S19, S20, S21, S22, S23 and S24.

**Comment 5**—As a last note, we think that in places the manuscript could benefit from a quick edit for English (probably a bit beyond what copy-editing might catch). We suggest that you run the manuscript by a critical colleague to help improve this aspect as this will help make the data accessible to our broad audience.

**Response 5**—We followed the advice from the editorial board and had sent the revised manuscript to professional language editing prior the resubmission. A proof of the language editing service is also enclosed in this resubmission.

**Response to the reviewers:**

**Reviewer #1:**
**Comment 1**—"It is unclear how the criteria for determining aged, non-diseased brains and AD brains were established. It is important for the criteria to be consistent across the four cohort studies to ensure accurate comparisons.", was not addressed in the revision though a supplemental table S1 was included. MY question is about how AD was determined in each of the four cohorts analyzed. If AD was defined differently, the analysis need be repeated based on the data with a consistent definition of AD cases.

**Response 1**—We apologize for mis-interpreting this comment during the first revision. While four cohorts of samples were included from Mathys et al. [1], Lau et al. [2], Nagy et al. [3], and Yang et al. [4], only two of them contained AD-related samples (i.e., Mathys et al. [1] and Lau et al. [2]) (Figure 2A).

According to the sample details extracted from the Mathys et al. study [1] **(Original publication: Supplementary Table 1)**, all their samples belonged to participants in the Religious Order Study (ROS) or the Rush Memory and Aging Project (MAP) (ROSMAP). Parameters they considered for categorizing samples into disease-affected (i.e., AD) and non-dementia (ND) cases were primarily based on the degree of classic disease pathologies manifested in the samples. These included the “global AD pathology burden (gpath)” (Average scores: ND=0.08 ± 0.08; AD=1.51 ± 0.74); “overall amyloid level (amyloid)” (Average scores: ND=0.03 ± 0.08; AD=6.46 ± 2.31) and the “neuritic plaque burden (plaq_n)” (Average scores: ND=0.01 ± 0.04; AD=1.79 ± 1.35). The definitions of these parameters are listed out clearly on the Rush Alzheimer’s Disease Centre (RADC) resource hub. Such obvious differences in the mean ± SD values in each of these domains indicated that the pathology-based classification of samples as AD versus ND were clear-cut without ambiguity.

On the other hand, according to the sample details extracted from the Lau et al study [2] study **(Original publication: SI Appendix, Table S1)**, their ND and AD samples were also defined based on the huge differences in disease-related pathologies, these included the “Age-related plaque scores” (i.e., ND=0/A; AD=B/C; Plaque load severity _Low to High_ = A 🡪 B 🡪 C; 0 = nil) and “Braak tangle stage” (i.e., ND= ≤ 2; AD= ≥ 4).

As hinted from the respective values in both studies, the control ND cases were defined based on absence of pathologies whereas the AD cases were defined based on the undoubted manifestations of heavy amyloid load and neuritic plaque burden. These details are now added back to the Methods and Materials section.

**Reviewer #2:**
**Comment 1—**Abstract lines 25-30 is somewhat contradictory where the use of "substantial" is used to indicate that the process is common, but the following sentences describes these cells as "rare existences" and "rare cell populations." Remove the word "substantial" from line 25 as it is not needed, suggested edit, "Mounting evidence indicate that terminally differentiated neurons in the brain may re-commit to a cell cycle-like process under both normal aging and disease affected conditions."

**Response 1**—We thank the reviewer’s suggestion, and the sentence is amended accordingly in the revised manuscript (Lines 26-27).

**Comment 2**—Line 83: Identification "of" cell cycle re-engaging neurons in human brain snRNA-seq datasets

**Response 2**— The wordings are amended accordingly in revised manuscript (Line 81).

**Comment 3**—Line 130 is a bit confusing - needs editing for clarification.

**Response 3—**In response to the comment, we replaced the original sentence with “Since cell cycle is an ordered sequence of events…” in the revised manuscript (Line 128).

**Comment 4**—Line 151, suggest deleting the word "harvested."

**Response 4**—The word “harvested” is deleted in the revised manuscript.

**Comment 5**—Line 208, replace "lost" with "loss."

**Response 5**— The wordings are amended accordingly in revised manuscript (Line 203).

**Comment 6**—Line 210: neuron should be "neurons as"

**Response 6**— The wordings are amended accordingly in revised manuscript (Line 205).

**Comment 7**—Line 221: "Considered" should be "Considering."

**Response 7**— The wordings is no longer existing in the revised manuscript after language editing.

**Comment 8**—Line 527: To note, the study (Dehkordi et al 2021, Nature Aging; PMID: 35531351) also identified senescent excitatory neurons by evaluating the datasets evaluated here. Adding this reference would strengthen the conclusions presented here.

**Response 8**—The reference [88] is now added to the revised manuscript (Line 520).

**Comment 9**—Line 546: The study (Riessland et al 2018, Cell Stem Cell; PMID: 31543366) also found senescent dopaminergic neurons. Adding this reference will strengthen the interpretations from the PD dataset presented here.

**Response 9**—The reference [89] is now added to the revised manuscript (Line 539).

**Reviewer #3:**

**Comment 1**—I would like to emphasize the authors have dedicated effort to address most of the comments and critiques raised from the previous version of the manuscript. After considering their responses, I still believe that more can be done to address arm-level aneuploidies **or remove it** as it might be argued the authors are over-reaching.

I understand and appreciate the authors have studied SMART-seq data to follow up, but this was not conclusive. I urge the authors to instead leverage 10x sequences, that they have already analyzed to call genetic variants from pooled reads and determine if candidate regions have shown **loss of heterozygosity** compared to other region/candidate genes. This might be done in a similar fashion to study GWAS arrays **leveraging B-allele frequency** to detect aneuploidies in immortalized cell lines.

**Response 1**—We thank the reviewer’s thoughtful comment and here we conducted further analysis. With reference to the development of copy number variation detection methods in single cell transcriptomics [5-9], the method of XClone that based on the integration of read depth signals and allelic imbalance using B-allele frequency (BAF) of heterozygous variant was adopted [10] for the further analyses on the potential events of loss of heterozygosity (LOH) at target gene regions. We first validated the robustness of XClone in a sample of triple-negative breast cancer which significantly revealed positive signals of copy number variations (CNV) and imbalanced allele frequency in cell clones A and B as compared to the normal cells **(Response Figure 1A)**. By then, ND (N = 3) and AD samples (N = 3) extracted from the Lau et al. dataset [2] which contained the higher coverages of read depth were utilized to detect potential LOH at the target gene regions **(Response Figure 1B-C)**. One first obvious result was that despite with distinct read depth statistics, both results from the XClone and inferCNV revealed consistent CNV score profiles in excitatory neurons of the late senescent (LS) group versus those in other layers **(Response Figure 1B-C, Upper panels)**. However, in contrast to the results of the breast cancer samples **(Response Figure 1A),** no allelic imbalance was found among the excitatory neurons in the brain regardless of the positive CNV scores **(Response Figure 1B-C, Bottom panels).**

As an attempt to explain these observations, we reasoned that the LOH occurs in tumor cells could be an outcome of a serious of erroneous DNA duplication and segregations events accumulated in uncontrolled cell proliferations [11]. In the contrary, mature excitatory neurons are by default post-mitotic in nature and even when there is an attempt to re-initiate the cell cycle process, an immediate senescent-like response is ensured to permanently arrest these cells [12, 13], and hence these cells exhibited almost zero possibility in losing their allelic balance. As for the emergence of CNVs, these events could be arisen by any homologous recombination (HR) events between repeated sequences (recurrent CNVs) or by non-homologous recombination mechanisms that occur throughout the genome (non-recurrent CNVs), both of which could be executed without completing a full round of cell cycle [14].

Therefore, we agree with the Reviewer that the term “arm-level aneuploidies” shall be removed to prevent any misconception of the findings in the revised manuscript. The related codes used to generate the BAM files and the CNV/LOH heatmap for each sample are available in: <https://zenodo.org/doi/10.5281/zenodo.10604562> .

**Response Figure 1:** (A) Estimation of copy number variants and loss of heterogeneity among different cell clones in a triple-negative breast cancer sample. (B-C) Estimation of copy number variants (upper panel) and loss of heterogeneity (bottom panel) by XClone algorithm among all excitatory neurons from randomly selected (B) non-dementia sample and (C) Alzheimer’s disease cases of the Lau et al. dataset [2] .

**References:**

1.	Mathys H, Davila-Velderrain J, Peng Z, Gao F, Mohammadi S, Young JZ, et al. Single-cell transcriptomic analysis of Alzheimer's disease. Nature. 2019;570(7761):332-7. Epub 2019/05/03. doi: 10.1038/s41586-019-1195-2. PubMed PMID: 31042697; PubMed Central PMCID: PMCPMC6865822.

2.	Lau SF, Cao H, Fu AKY, Ip NY. Single-nucleus transcriptome analysis reveals dysregulation of angiogenic endothelial cells and neuroprotective glia in Alzheimer's disease. Proc Natl Acad Sci U S A. 2020;117(41):25800-9. Epub 2020/09/30. doi: 10.1073/pnas.2008762117. PubMed PMID: 32989152; PubMed Central PMCID: PMCPMC7568283.

3.	Nagy C, Maitra M, Tanti A, Suderman M, Theroux JF, Davoli MA, et al. Single-nucleus transcriptomics of the prefrontal cortex in major depressive disorder implicates oligodendrocyte precursor cells and excitatory neurons. Nat Neurosci. 2020;23(6):771-81. Epub 2020/04/29. doi: 10.1038/s41593-020-0621-y. PubMed PMID: 32341540.

4.	Yang AC, Kern F, Losada PM, Agam MR, Maat CA, Schmartz GP, et al. Dysregulation of brain and choroid plexus cell types in severe COVID-19. Nature. 2021;595(7868):565-71. Epub 2021/06/22. doi: 10.1038/s41586-021-03710-0. PubMed PMID: 34153974; PubMed Central PMCID: PMCPMC8400927.

5.	Fan J, Lee HO, Lee S, Ryu DE, Lee S, Xue C, et al. Linking transcriptional and genetic tumor heterogeneity through allele analysis of single-cell RNA-seq data. Genome Res. 2018;28(8):1217-27. Epub 2018/06/15. doi: 10.1101/gr.228080.117. PubMed PMID: 29898899; PubMed Central PMCID: PMCPMC6071640.

6.	Gao R, Bai S, Henderson YC, Lin Y, Schalck A, Yan Y, et al. Delineating copy number and clonal substructure in human tumors from single-cell transcriptomes. Nat Biotechnol. 2021;39(5):599-608. Epub 2021/01/20. doi: 10.1038/s41587-020-00795-2. PubMed PMID: 33462507; PubMed Central PMCID: PMCPMC8122019.

7.	Gao T, Soldatov R, Sarkar H, Kurkiewicz A, Biederstedt E, Loh PR, et al. Haplotype-aware analysis of somatic copy number variations from single-cell transcriptomes. Nat Biotechnol. 2023;41(3):417-26. Epub 2022/09/28. doi: 10.1038/s41587-022-01468-y. PubMed PMID: 36163550; PubMed Central PMCID: PMCPMC10289836.

8.	Patel AP, Tirosh I, Trombetta JJ, Shalek AK, Gillespie SM, Wakimoto H, et al. Single-cell RNA-seq highlights intratumoral heterogeneity in primary glioblastoma. Science. 2014;344(6190):1396-401. Epub 2014/06/14. doi: 10.1126/science.1254257. PubMed PMID: 24925914; PubMed Central PMCID: PMCPMC4123637.

9.	Serin Harmanci A, Harmanci AO, Zhou X. CaSpER identifies and visualizes CNV events by integrative analysis of single-cell or bulk RNA-sequencing data. Nat Commun. 2020;11(1):89. Epub 2020/01/05. doi: 10.1038/s41467-019-13779-x. PubMed PMID: 31900397; PubMed Central PMCID: PMCPMC6941987.

10.	Huang R, Huang X, Stegle O, Huang Y. Robust analysis of allele-specific copy number variations from scRNA-seq data with XClone. 2023:2023.04.03.535352. doi: 10.1101/2023.04.03.535352 %J bioRxiv.

11.	Pedersen BS, De S. Loss of heterozygosity preferentially occurs in early replicating regions in cancer genomes. Nucleic Acids Res. 2013;41(16):7615-24. Epub 2013/06/25. doi: 10.1093/nar/gkt552. PubMed PMID: 23793816; PubMed Central PMCID: PMCPMC3763542.

12.	Chow HM, Shi M, Cheng A, Gao Y, Chen G, Song X, et al. Age-related hyperinsulinemia leads to insulin resistance in neurons and cell-cycle-induced senescence. Nat Neurosci. 2019;22(11):1806-19. Epub 2019/10/23. doi: 10.1038/s41593-019-0505-1. PubMed PMID: 31636448.

13.	Sun JK, Wu D, Wong GC, Lau TM, Yang M, Hart RP, et al. Chronic alcohol metabolism results in DNA repair infidelity and cell cycle-induced senescence in neurons. Aging Cell. 2023;22(2):e13772. Epub 2023/01/24. doi: 10.1111/acel.13772. PubMed PMID: 36691110; PubMed Central PMCID: PMCPMC9924945.

14.	Hastings PJ, Lupski JR, Rosenberg SM, Ira G. Mechanisms of change in gene copy number. Nat Rev Genet. 2009;10(8):551-64. Epub 2009/07/15. doi: 10.1038/nrg2593. PubMed PMID: 19597530; PubMed Central PMCID: PMCPMC2864001.
